# Bulk Processing of Multi-Temporal Modis Data, Statistical Analyses and Machine Learning Algorithms to Understand Climate Variables in the Indian Himalayan Region

**DOI:** 10.3390/s21217416

**Published:** 2021-11-08

**Authors:** Mohd Anul Haq, Prashant Baral, Shivaprakash Yaragal, Biswajeet Pradhan

**Affiliations:** 1 Department of Computer Science, College of Computer Science and Information Sciences, AL-Majmaah 11952, Saudi Arabia; 2 Geographic Information Systems, NIIT University, Neemrana 301705, Rajasthan, India; prashant.baral@st.niituniversity.in; 3 Esri India Technologies Ltd., Noida 201301, Uttar Pradesh, India; shivaprakash.ssy@gmail.com; 4 The Centre for Advanced Modelling and Geospatial Information Systems (CAMGIS), Faculty of Engineering and IT, University of Technology Sydney, Sydney, NSW 2007, Australia; 5 Center of Excellence for Climate Change Research, King Abdulaziz University, P.O. Box 80234, Jeddah 21589, Saudi Arabia; 6 Earth Observation Centre, Institute of Climate Change, Universiti Kebangsaan Malaysia, Bangi 43600, Selangor, Malaysia

**Keywords:** machine learning, remote sensing, global climate data, MODIS, Uttarakhand, Himalaya

## Abstract

Studies relating to trends of vegetation, snowfall and temperature in the north-western Himalayan region of India are generally focused on specific areas. Therefore, a proper understanding of regional changes in climate parameters over large time periods is generally absent, which increases the complexity of making appropriate conclusions related to climate change-induced effects in the Himalayan region. This study provides a broad overview of changes in patterns of vegetation, snow covers and temperature in Uttarakhand state of India through bulk processing of remotely sensed Moderate Resolution Imaging Spectroradiometer (MODIS) data, meteorological records and simulated global climate data. Additionally, regression using machine learning algorithms such as Support Vectors and Long Short-term Memory (LSTM) network is carried out to check the possibility of predicting these environmental variables. Results from 17 years of data show an increasing trend of snow-covered areas during pre-monsoon and decreasing vegetation covers during monsoon since 2001. Solar radiation and cloud cover largely control the lapse rate variations. Mean MODIS-derived land surface temperature (LST) observations are in close agreement with global climate data. Future studies focused on climate trends and environmental parameters in Uttarakhand could fairly rely upon the remotely sensed measurements and simulated climate data for the region.

## 1. Introduction

A good understanding of changes related to temperature, areas covered with snow and vegetation is a preliminary step towards developing meaningful conclusions associated with the variations in these environmental components [1,2]. The knowledge is an important guide to explore the interconnectedness among these environmental components and the influence of changes in one component affecting other environmental variables [3,4,5]. There are examples of studies where remote sensing observations along with machine learning algorithms have been used to analyse environmental components over wide-ranging areas [6,7].

Some previous studies have mentioned the changes in environmental components in the Uttarakhand state of the north-western Himalayan region [8,9]. Most of the studies in this state have mentioned some local changes or considered only one or two environmental parameters [10,11]. Examples of studies where remotely sensed data in combination with machine learning algorithms are applied to study environmental components in the north-western Himalayan region of Uttarakhand are not pronounced [12].

Aqua Earth Observation System and Terra Earth Observation System monitor the Earth’s surface using a Moderate Resolution Imaging Spectroradiometer (MODIS) sensor system, the principal instrument in these satellites [13]. This instrument records data to analyse several environmental variables such as greenness [14], areas covered with snow [15], Earth’s surface temperature [16], and many others [17]. These grids are available for different intervals of time and in different grid cell sizes. MODIS grids have remained largely successful in providing information about global as well as regional changes in environmental parameters over time [18,19,20].

There are several global climate data set available for the study of past, present and future climate conditions [21]. These data set could be a collection of field based records or results of numerical simulations or a combination of both. Availability of these climate data set provide with an opportunity to understand past, present and future climate conditions. As there are a number of global climate data sets available for analysis, using multiple data set in a single study helps identify those data set that are more accurate in representing actual ground conditions. MODIS data sets are available since 2001 and therefore, application of MODIS data set can help understand the climate of the recent past as well as the present climate conditions. However, there are climate data set that can provide information about climatic variables since 1970 [22]. Therefore, combination of MODIS climate data with global climate data can help study not only the present climate as well as the climate of the recent past but also climate conditions before several decades.

To establish an all-inclusive understanding about variations in environmental components in different elevation zones of Uttarakhand in India, this study uses 17 years of gridded data available from MODIS sensor to analyse variations in extents of snow and vegetation and distribution of land surface temperature. A large volume of remotely sensed data has been bulk processed, and machine learning operations have been additionally applied to discover changes in environmental components. The interconnectedness among the analysed environmental parameters has been realized for different elevation zones within the state of Uttarakhand. Periodic changes in the environmental components annually as well as for different seasons within a year are observed. To authenticate findings from remote sensing observations, field-based records available from meteorological stations are used. In addition, machine learning operations have been applied for predicting values of environmental variables. Consequently, the usage of remotely sensed data in combination with machine learning and statistical operations has been assessed to reflect upon the ongoing environmental changes in the region. This study also aims to provide information about the degree of agreement of MODIS climate data with the global climate data set available from different sources. This information can be useful in the application of MODIS data, in combination with other global climate data, to study different regions in the remote Himalayan region, where data from the past are usually unavailable.

Localized studies focused on the assessment of snow cover, vegetation and land surface temperatures in particular places of Uttarakhand are essential to understand the variability in these environmental attributes. However, these studies do not help understand the altitudinal variations in these parameters. Similarly, these studies are carried out for a specific time period, making it difficult to understand changes occurring over larger time scales. Additionally, there are few studies that explore the variability among the environmental parameters [9]. This study attempts to provide detail information about the variations in multiple environmental factors over the entire state of Uttarakhand, which is an essential preliminary step in understanding the interrelationship among environmental variables. Additionally, this study explores the application potential of neural networks and deep learning methods for prediction of environmental variables, which is otherwise an unusual practice for this north-western Himalayan state. This study is possibly the first to examine the changes in more than two environmental variables over entire Uttarakhand for almost two decades using a combination of bulk processing of remotely sensed data set and application of machine learning and deep learning methods.

## 2. Study Area

**Figure 1:**
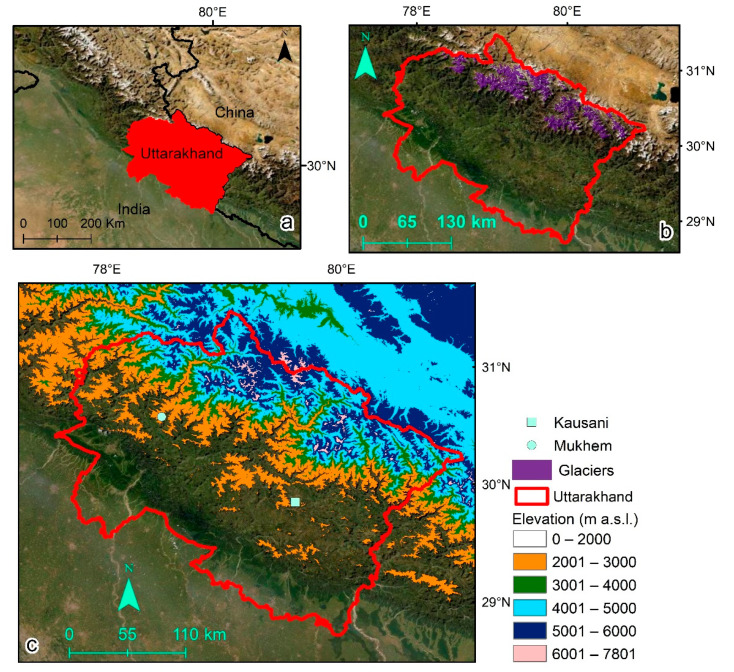
(**a**) Area in red shows Uttarakhand inside the map of India. (**b**) Political boundary of Uttarakhand is delineated in red. All glaciers located within the political boundary of Uttarakhand are shown in purple. Glacier boundaries are accessed through Randolph Glacier Inventory [25]. Base-map layer [26] fills the background. (**c**) Multiple colour legends highlight different elevation classes in Uttarakhand. Two locations, Kausani and Mukhem, in Uttarakhand are shown.

The region of Uttarakhand in India expands from 28.71° N in the north to 31.47° N in the south and from 77.56° E in the west to 81.02° E in the east. The north-western Himalayan state almost occupies an area about 53,500 km^2^. The lowest and highest elevation in the state is recorded around 200 m a.s.l. and 7800 m a.s.l., respectively (Figure 1). Precipitation distribution in Uttarakhand is majorly controlled by Asian monsoon as well as the Westerlies [23]. The monsoon precipitation generally begins in July and continues until the end of September, whereas the precipitation from Westerlies is received during winter months. Since there are mountains in low elevation regions in Uttarakhand, sub-temperate climate prevails in such areas. The climate turns temperate as we move towards the mountains in the high elevation regions [24].

## 3. Data

### 3.1. MODIS Grids

MOD10A2 grid, available as a composite product representing average conditions of snow extent, is a Terra grid and has a spatial resolution of 500 m [15]. MOD11A2 grid, also available as a composite product representing average conditions of land surface temperature, is another Terra grid with a spatial resolution of 1000 m [16]. Additionally, MOD13A1 grid, also available as a composite product representing average conditions of vegetation or greenness cover, is another Terra grid with a spatial resolution of 500 m [14]. MOD10A2 and MOD11A2 are 8-day composites, whereas MOD13A1 is a 16-day composite product.

MODIS grids, MOD10A2, MOD11A2 and MOD13A1 are used in this study to obtain information related to snow coverage, temperature distribution and Normalized Difference Vegetation Index (NDVI), respectively (Table 1).

### 3.2. Elevation Grid

Elevation grid, SRTMGL1 V003 [27] provides the elevation data for analysis. This grid is a global 1 arc second (~30 m) grid generated through the Shuttle Radar Topography Mission (SRTM) mission.

### 3.3. Meteorological Records

Field-based meteorological records available from local stations located in Kausani as well as Mukhem are incorporated in this study.

### 3.4. Climate Data

Climate data from WorldClim 2.0 [22], CHELSA (Climatologies at high resolution for the earth’s land surface areas) [28] and MIROC-ES2L (Model for Interdisciplinary Research on Climate, Earth System version 2 for Long-term simulations) [29] have been used for comparisons among remotely sensed observations and modelled climate data set. WorldClim 2.0 provides one of the highest resolution global climate data for 1970–2000. The data has been generated using records from ground-based meteorological records. CHELSA has been generated through statistical downscaling using global reanalysis data. Four bioclimatic variables from these three data sets were considered in this study: BIO1, BIO10, BIO12 and BIO16. In this study, BIO1 has been referred to as Mean Annual Air Temperature (MAAT hereafter), BIO10 is referred to as Mean Temperature of Warmest Quarter (T-WQ hereafter), BIO12 is referred to as Annual Precipitation (AnP hereafter) and BIO16 is referred to as Precipitation of Wettest Quarter (P-WQ hereafter). The maximum, minimum and mean values of these four bioclimatic variables obtained from the three global climate data set for the 10 elevation sections within Uttarakhand were estimated and these values were compared with results obtained from MODIS data set.

Data sets used in this study, except for the data from the meteorological records, are all freely available. Use of freely available data sets makes this study simpler and easy to reproduce. Regional analysis using freely available data are few, therefore, this study is intended to produce an example for a reasonable analysis using freely available data sets for the western Himalayan region.

## 4. Methods

**Figure 2:**
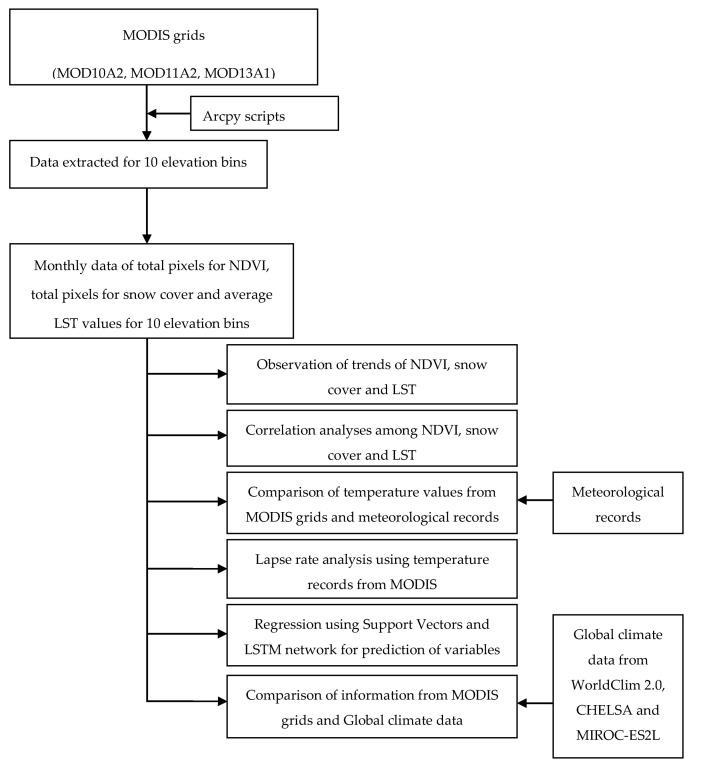
Methodological flow chart to explain step wise process followed in this study.

Flow diagram (Figure 2) summarizes the methods used in this study.

### 4.1. MODIS Data

MODIS grids were downloaded for the years 2001–2017. The state of Uttarakhand was partitioned into 10 different areas based on elevation. Regions with elevation lower than 2000 m a.s.l. and higher than 6000 m a.s.l. were classified into two (2) areas. Regions with elevation from 2000 m a.s.l. to 6000 m a.s.l. were classified into eight (8) areas, with each area having an elevation difference of 500 m. These partitions were made using SRTMGL1 V003 in GIS environment.

ArcPy, an arcgisscripting module for performing geospatial analyses using Python. ArcPy scripts, were written to operate with downloaded MODIS grids and withdraw subset layers. ArcPy scripts are able to perform necessary operations related to layer scaling and layer extraction. Binary classification of grids was followed to withdraw grids for each elevation area and hence 10 grids were generated. Temporal inspections were executed using monthly observations. For NDVI, a threshold value of 0.3, NDVI ≥ 0.3, was accepted. The resulting outputs include monthly observations of temperature, vegetation and snow cover from 2001 to 2017. A similar workflow was recently applied to observe environmental components in Himachal Pradesh, India [30].

### 4.2. Observation of Trends

Monotonic patterns and sudden fluctuations in numerical values of climate parameters for the years 2001–2017 were observed through Mann–Kendall tests [31,32]. Computation of Sen’s slope [33] also contributed to the observation of tends of the parameters. Statistical output (*S*) from Mann–Kendall was obtained through time series of monthly observations of climate parameters (Equation (1)). Variance for the same set of data *VAR*(*S*) was calculated (Equation (2)) and this was followed by Standardized test *Z* (Equation (3)) [34].

(1) $$\begin{array}{l} S=\overset{n-1}{\underset{i=1}{\sum }}\overset{n}{\underset{j=i+1}{\sum }}Sgn\left(X_{j}-X_{i}\right),\\ Sgn\left(X_{j}-X_{i}\right)=\{\begin{array}{c} +1,>\left(X_{j}-X_{i}\right)\\ 0,=\left(X_{j}-X_{i}\right)\\ -1,<\left(X_{j}-X_{i}\right), \end{array} \end{array}$$

(2) $$VAR\left(S\right)=\frac{1}{18}\left[n\left(n-1\right)\left(2n+5\right)-\overset{q}{\underset{p=1}{\sum }}t_{p}\left(t_{p}-1\right)\left(2t_{p}+5\right)\right],$$

(3) $$Z\;\{\begin{array}{c} \frac{S-1}{\sqrt{VAR\left(S\right)}}\;if\;S>0\;\\ 0\;if\;S=0\\ \frac{S+1}{\sqrt{VAR\left(S\right)}}\;if\;S<0. \end{array}$$

In Equation (1), *X_i_* and *X_j_* are terms representing the values of parameters that were chronologically placed. Similarly, in Equation (2), *n* indicates the total length of the time series, *t_p_* shows ties corresponding to the *p*th value, *q* shows total tied values. When the value of *Z* is positive; it signifies a variables trend that is increasing and when this value is negative; it indicates a decreasing trend. All equations were processed using XLSTAT and Microsoft Excel [35].

### 4.3. Correlation Analyses

Using monthly observations of climate parameters, Pearson correlation function was used to determine the nature of association among climate parameters. Accordingly, total cells in the NDVI grid layer and average temperature values for each month were correlated. Similarly, total cells in the NDVI grid layer and snow grid layer were correlated. Additionally, total cells in the snow grid layer and average temperature values were also correlated. This process was executed for the entire area of observation, as well as for the ten separate elevation sections. All computations were performed in International Business Machines Corporation’s Statistical Package for the Social Sciences (IBM SPSS) Statistics [36]. To estimate the nature of the relationship between climate data from MODIS and ground observations, linear correlation and linear regression among MOD11A2 values and values recorded at stations in Kausani and Mukhem were observed.

### 4.4. Lapse Rate

Monthly averages of MOD11A2 for different elevation sections were used to compute monthly, annual as well as seasonal lapse rates for Uttarakhand. Values of 8 elevation sections for the years 2001–2017 were used. Therefore, temperature values from 2000 m a.s.l. to 6000 m a.s.l. were considered. The averages of the lowermost and uppermost elevation of each section were computed and the monthly average of temperature for that elevation section was attributed to the average value of the lowermost and uppermost elevation. Consequently, 8 averages (2250, 2750, 3250, 3750, 4250, 4750, 5250 and 5750 m a.s.l.) corresponding to 8 elevation sections were generated. Using 8 averages for the elevation sections and their corresponding monthly temperature averages, linear regression was performed to obtain regression lapse rate. Thus, 204 monthly lapse rate values were computed, which were later used to generate annual lapse rate, seasonal lapse rate and lapse rate for each month.

### 4.5. Support Vector Regression

The regression is performed using a data set $\left\{(x_{1},y_{1}),....,(x_{\ell },y_{\ell })\right\}$ considered for training. In this data set, $x_{i}\subset R^{n}$ signifies the input having an output $y_{i}\subset R$ where *i* = 1, …, *n*. The term *n* signifies the data length [37,38]. The equation for regression is written as:

(4) f(x)=(w·Φ(x))+b

In the equation, $w\subset R^{n}$ as well as b⊂R. Additionally, Φ symbolizes transformation that is non-linear in nature and occurs from $R^{n}$ to a space that lies in higher dimension. Solving the equation provides the values for w and b.

Following this, x is determined when the risk from regression is reduced:

(5) $$R_{reg}(f)=C\sum_{i=0}^{\ell }\Gamma (f(xi)-yi)+\frac{1}{2}{\left\|w\right\|}^{2}$$

In the equation, Γ(·) denotes the cost function, C is a constant and w denotes a vector. The equation for the vector can be written as:

(6) $$w=\sum_{i=1}^{\ell }\left(\alpha_{i}-\alpha_{i}{}^{*})\Phi (x_{i}\right)$$

Replacing w as expressed in (6) in the Equation (4), we get:

(7) $$\begin{array}{cc} f(x) & =\sum_{i=1}^{\ell }\left(\alpha_{i}-\alpha_{i}{}^{*})(\Phi (x_{i})\cdot \Phi (x)\right)+b\\ & =\sum_{i=1}^{\ell }\left(\alpha_{i}-\alpha_{i}{}^{*})k(x_{i},x\right)+b \end{array}$$

As can be seen in Equation (7), *k* (*x_i_*, *x*) replaces the dot product. In the expression, *k* (*x_i_*, *x*) is the kernel function. Two kernels, linear as well as polynomial, were tested in this study.

The regression function was used for forecasting one climate variable using known conditions of the remaining two climate variables, and this appraisal was carried out for all 3 climate variables. For the training data, observations from 2001 to 2012 were selected and regression models were developed. These models predicted values of the climate variables from 2013 to 2017. Finally, predicted values were compared with the observations from MODIS grids to check the efficiency of the models.

### 4.6. Long Short-Term Memory (LSTM) Regression Analysis

Long Short-term Memory (LSTM) neural network is a special class of recurrent neural networks (RNN) [39] and has both special units and standard units. LSTM is mostly used for carrying out regression functions.

Scripts in Python were written and Keras library [40] was accessed to construct the neural network. Additionally, Tensorflow [41] was used for the model to process. Within the network, Sequential module helped the network to begin working and dense module assisted in the accumulation of required layers within the network. Scaling was performed and stochastic gradient descent method was used for running the network. Using all these combinations, results were simulated using 100 epochs.

Composition function in [42,43], outlines the LSTM network applied for the present analysis.

The network was used for forecasting one climate variable using known conditions for the remaining two climate variables, and this appraisal was carried out for all three climate variables. For the training data, observations from 2001 until 2012 were selected and LSTM models were developed. These models predicted values of the climate variables from 2013 until 2017. Finally, predicted values were compared with the observations from MODIS grids to check the efficiency of the models.

## 5. Results

### 5.1. Distribution of Snow Extent, Vegetation Area and Temperature

NDVI grid layer with NDVI ≥ 0.3, temperature and snow coverage in Uttarakhand provided information about these environmental variables within different elevation sections of Uttarakhand.

**Figure 3:**
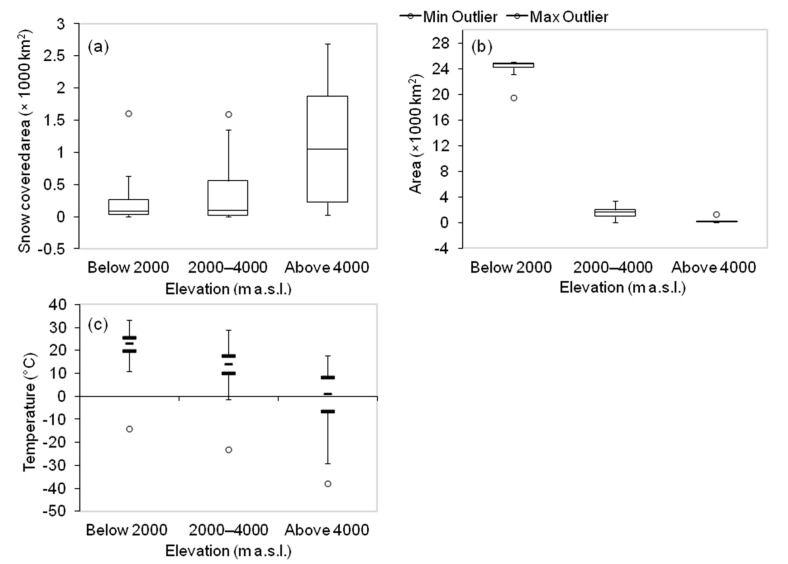
Boxplot displaying (**a**) area of NDVI grid layer ≥ 0.3 in different elevation zones, (**b**) area of snow cover in different elevation zones (**c**) temperature in different elevation zones in Uttarakhand. Circles show outliers and lines within boxes show median values.

Land areas occupying more than 24,000 km^2^ below the elevation of 2000 m a.s.l. is found to have vegetation cover in Uttarakhand state. Above 4000 m a.s.l., vegetation is negligibly present. From 2000 m a.s.l. to 4000 m a.s.l., vegetation cover is less than 4000 km^2^ (Figure 3). Snow occupied areas exist even below 2000 m a.s.l. Significant fluctuations in snow cover can be seen above 4000 m a.s.l. Median values for land surface temperature stay about 25 °C for areas lower than 2000 m a.s.l. Similarly, for areas within 2000 to 4000 m a.s.l., temperature values generally remain about 15 °C and for areas higher than 4000 m a.s.l., temperature drops to about 0 °C.

**Figure 4:**
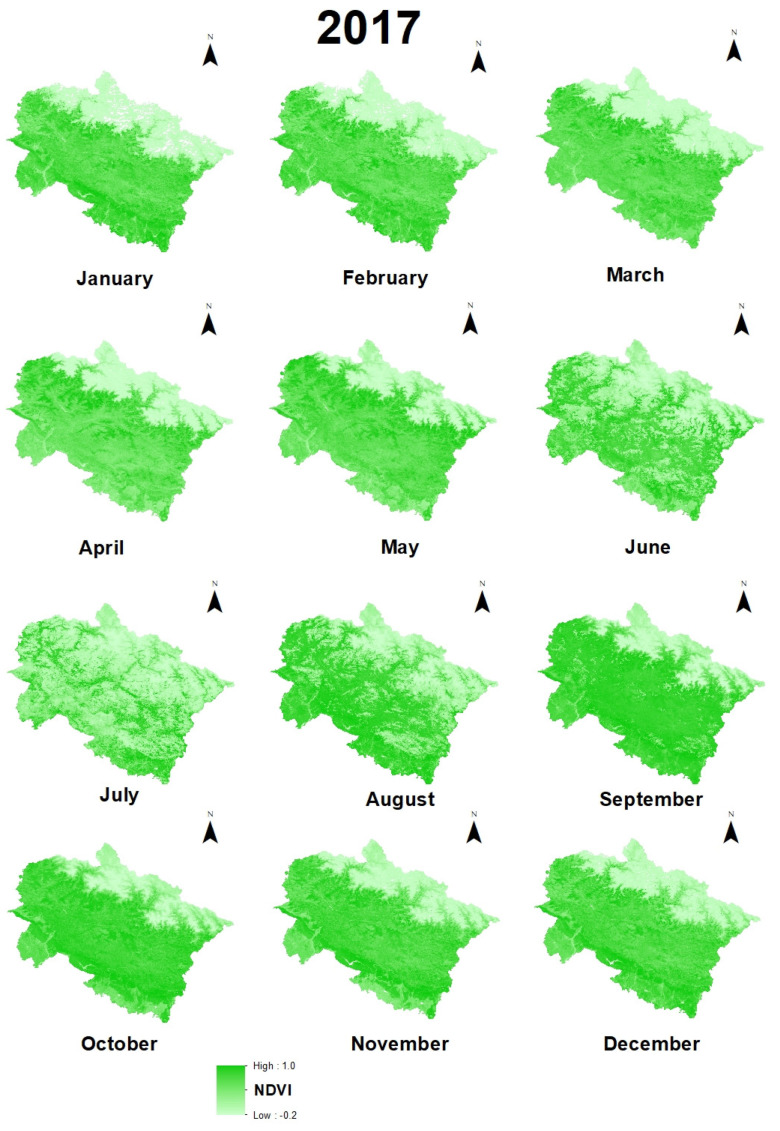
Monthly fluctuations of vegetation cover in Uttarakhand in 2017.

**Figure 5:**
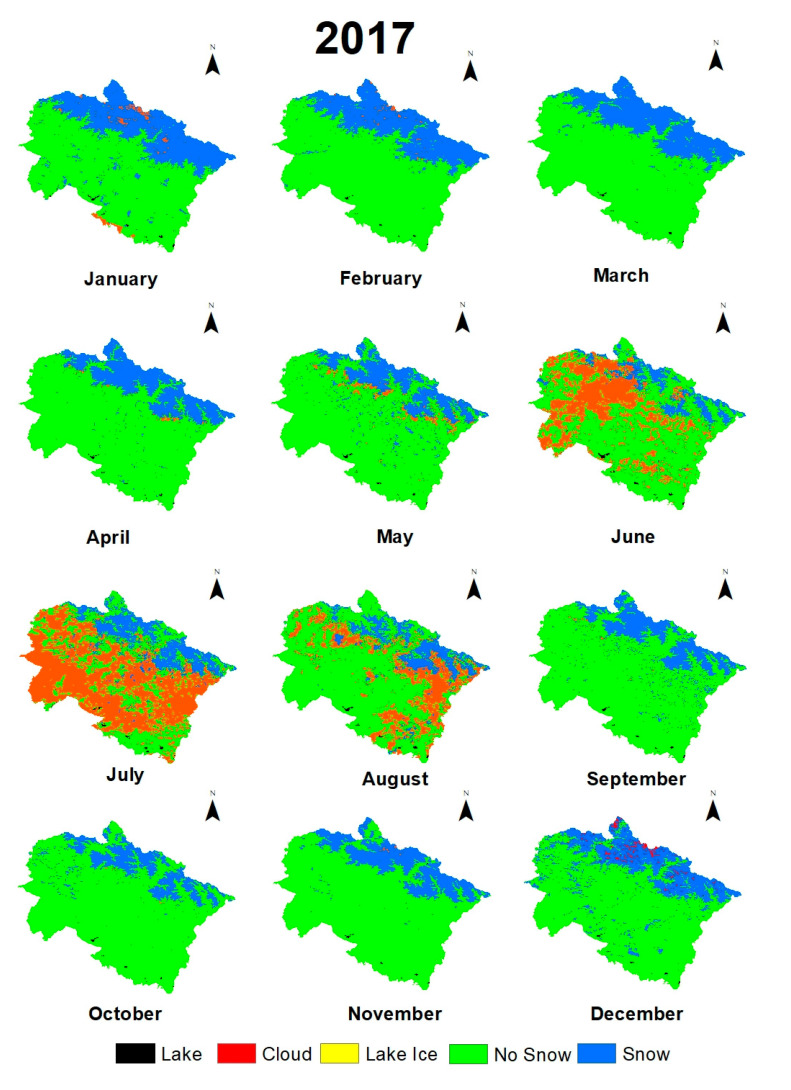
Monthly fluctuations of snow cover in Uttarakhand in 2017.

**Figure 6:**
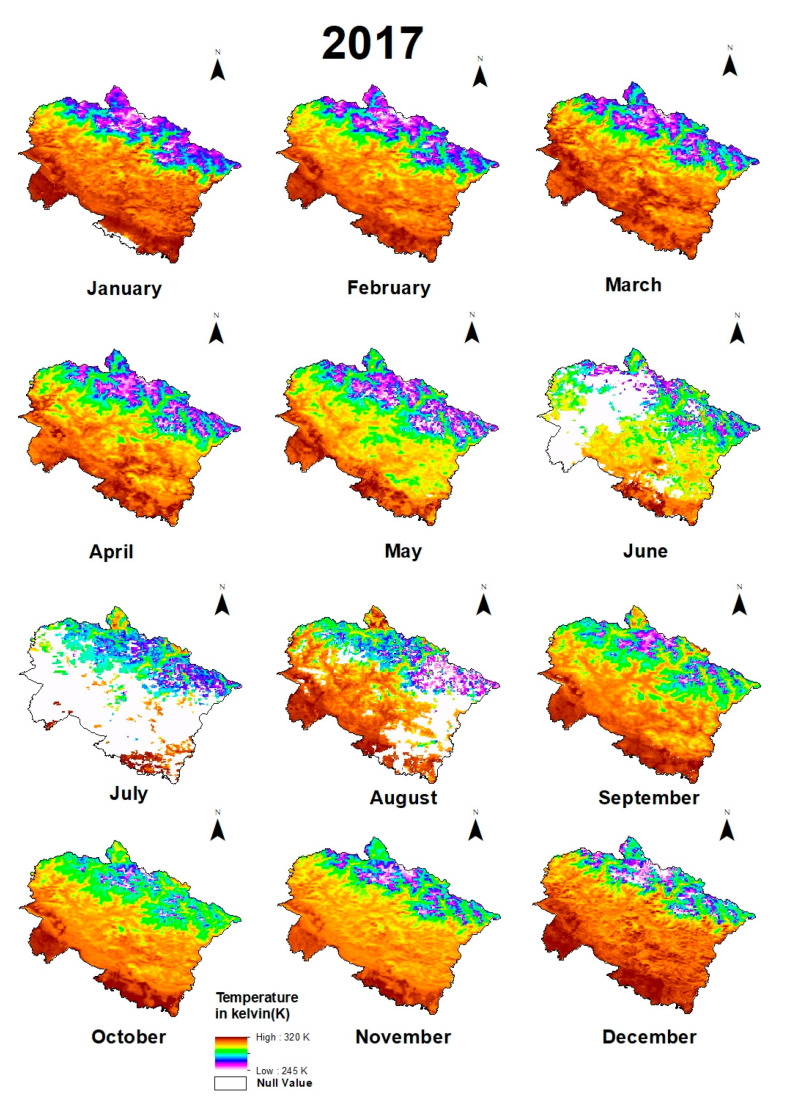
Monthly fluctuations of temperature in Uttarakhand in 2017.

Maps of monthly variations of environmental variables in 2017 (Figure 4, Figure 5 and Figure 6) and previous months and statistical calculations provided an understanding of these variations.

**Figure 7:**
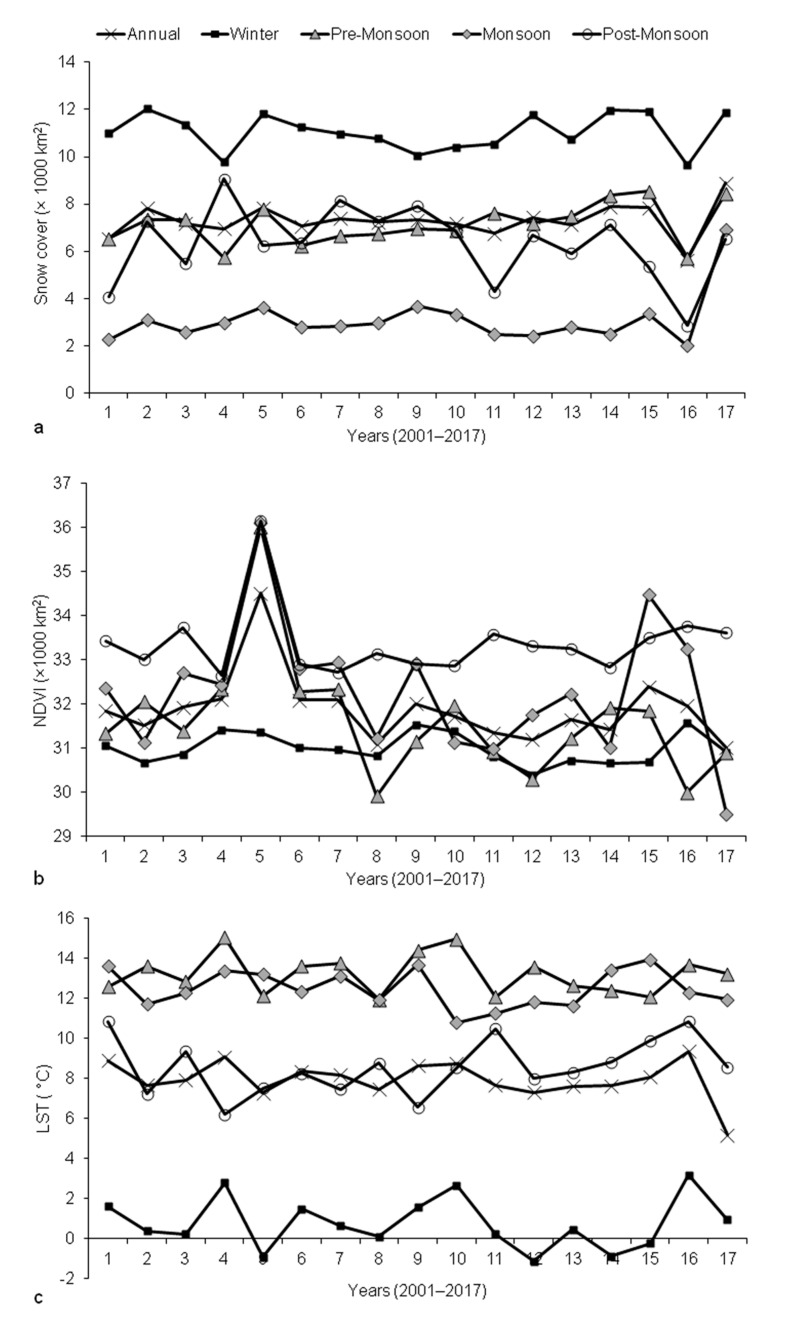
Changes in (**a**) snow cover, (**b**) vegetation covers with NDVI ≥ 0.3 and (**c**) average temperature occurring annually and in different seasons in Uttarakhand.

A vertical rise related to vegetation covers was observed for the years 2004–2005 (Figure 7). Less snowfall in 2004 and hence lack of snow-covered areas could be responsible for the increase in vegetation covers. Quite reasonably, much lower temperatures were observed in winter and relatively higher temperatures were observed in pre-monsoon compared to other seasons. The region has the least snow cover during monsoon and the most during winter.

### 5.2. Trend Analysis

Observations of monotonic trends and sudden fluctuations in patterns of environmental variables for the entire state of Uttarakhand contained several interesting outputs with a confidence level around or greater than 85%. These outputs suggested that Uttarakhand has been experiencing an increase in annual snow covers for the years 2001–2017. This increase is particularly visible for the pre-monsoon season. Monthly observations showed that this is true, especially for the months from May to July. Consequently, areas of vegetation have been decreasing for the pre-monsoon periods and this can be seen clearly for the months of June and July. When temperature is considered, no significant trends were observed.

Observation of monotonic trends and sudden fluctuations in patterns of environmental variables for the different elevation sections of Uttarakhand also contained several interesting outputs with a confidence level around or greater than 85%. Snow covers were found to increase for the region beyond 4000 m a.s.l. through pre-monsoon. For the areas lying from 2000 m a.s.l. until 4000 m a.s.l., post-monsoon vegetation covers have increased. Pre-monsoon temperatures in areas lower than 2000 m a.s.l. have increased.

### 5.3. Correlation Analyses

Correlation function showed strong connections among the variables for the elevation range 3000–4000 m a.s.l. Quite justifiably, for almost all elevation sections, average temperatures and snow covers have a pronounced negative relationship and this also applies to the relationship among vegetation covers and snow covers.

### 5.4. MODIS LST and Station Data

#### 5.4.1. Mukhem Station

**Figure 8:**
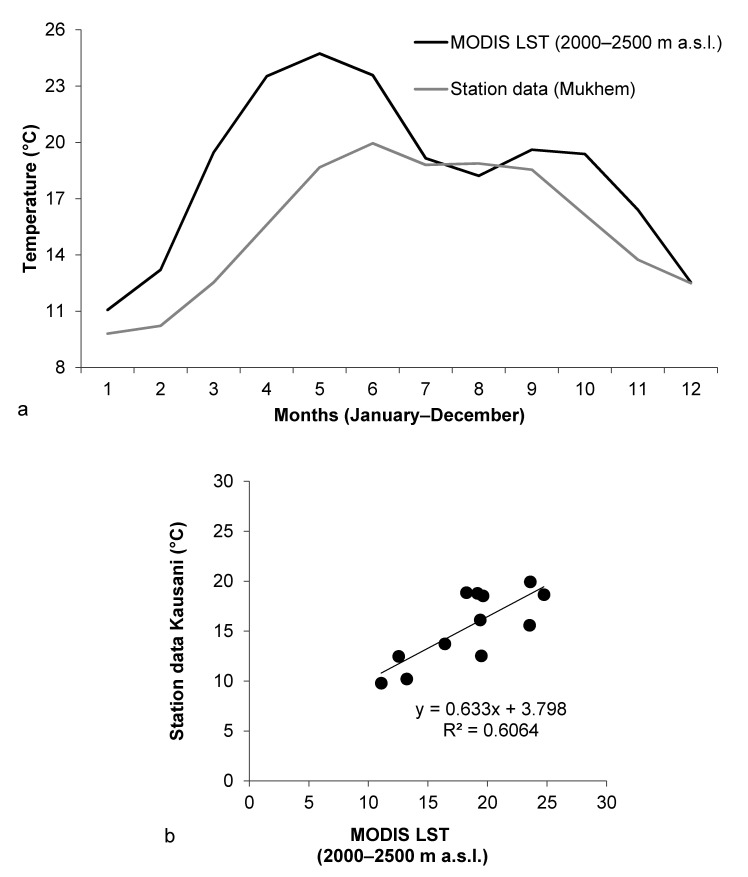
(**a**) Linear curve for the average value of maximum temperature for each month drawn using the temperature records at Mukhem for the years 2001–2008 and linear curve for the mean values of temperature for each month obtained using MODIS grid for the years 2001–2008 for the elevation ranging from 2000 to 2500 m a.s.l. (**b**) Scatter plot drawn using the average value of maximum temperature for each month based on temperature records at Mukhem for the years 2001–2008 and the mean values of temperature for each month obtained using MODIS grid for the years 2001–2008 for the elevation ranging from 2000 to 2500 m a.s.l.

Temperature data from MODIS temperature grid used in this study and temperature records from the station in Mukhem in Uttarakhand were compared (Figure 8). A scatter plot to compare the data from Mukhem and MODIS grid information for the elevation section 2000–2500 m a.s.l. was drawn and the equation for linear fit was obtained. The regression equation showed that the R-squared value remained at around 0.60 which indicated that the two data were fairly related.

#### 5.4.2. Kausani Station

**Figure 9:**
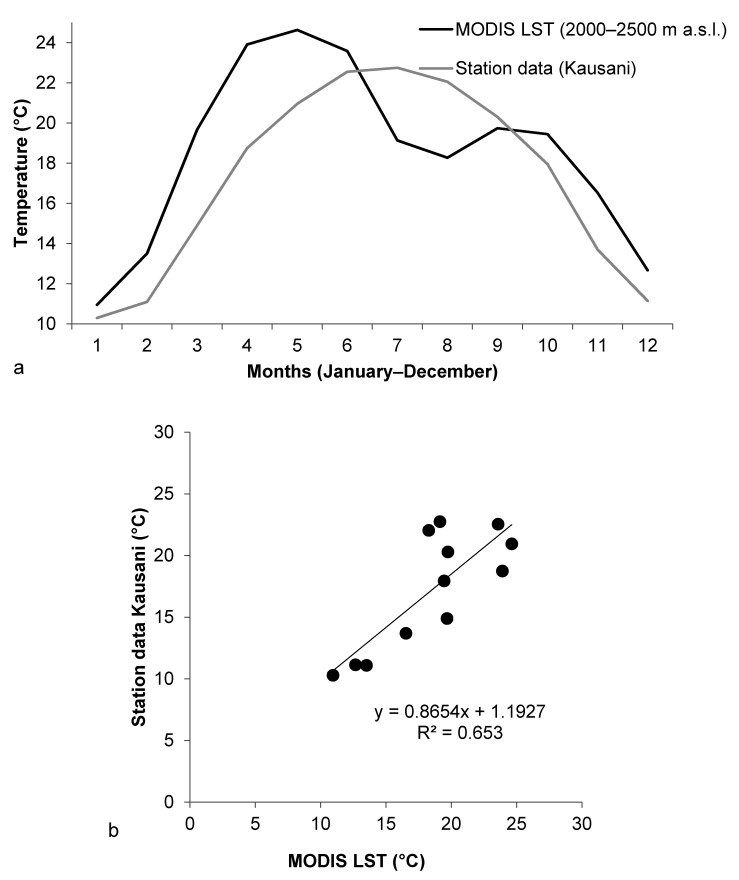
(**a**) Linear curve for the average value of maximum temperature for each month drawn using the temperature records at Kausani for the years 2001–2009 and linear curve for the mean values of temperature for each month obtained using MODIS grid for the years 2001–2009 for the elevation ranging from 2000 to 2500 m a.s.l. (**b**) Scatter plot drawn using the average value of maximum temperature for each month based on temperature records at Kausani for the years 2001–2009 and the mean values of temperature for each month obtained using MODIS grid for the years 2001–2009 for the elevation ranging from 2000 to 2500 m a.s.l.

Temperature data from MODIS temperature grid used in this study and temperature records from the station in Kausani in Uttarakhand were compared (Figure 9). A scatter plot to compare the data from Kausani and MODIS grid information for the elevation section 2000–2500 m a.s.l. was drawn and the equation for linear fit was obtained. The regression equation showed that the R-squared value remained at around 0.65 which indicated that the two data were fairly related.

### 5.5. Lapse Rate Analysis

**Figure 10:**
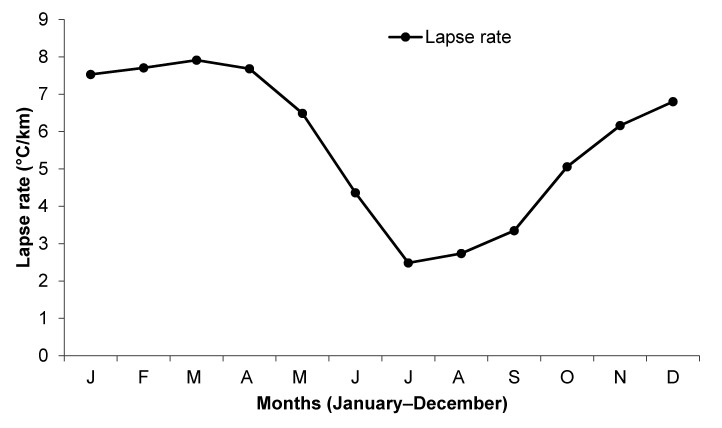
Distribution of the lapse rate values in Uttarakhand for all months in a year for the elevation ranging from 2000 to 6000 m a.s.l.

Lapse rate calculations showed that the yearly value of lapse rate when the entire area of Uttarakhand state is considered is 5.69 °C/km. Similarly, the value of lapse rate for the monsoon period is 2.86 °C/km, the post-monsoon period is 5.61 °C/km, the winter period is 7.49 °C/km and the pre-monsoon period is 6.18 °C/km. The lapse rate is highest during the winter and lowest during the monsoon period. A distinct periodical change in the value of lapse rate is seen for the different periods in a year in Uttarakhand (Figure 10). A distinct low lapse rate during monsoon could be mainly due to the continuous existence of clouds during the monsoon period that minimize the effect of solar radiation upon temperature distribution on the Earth’s surface.

### 5.6. Support Vector Regression Analysis

Regression using support vectors, in the absence of scaling operations, displayed that forecasting of temperature is the most accurate when linear kernel is used in the regression and the values for both constant and gamma function are fixed at 100. Similarly, forecasting of snow covers is the most accurate when polynomial kernel is used in the regression and the value for constant is fixed at 100 and gamma function is defined as “auto”. In the same way, forecasting of NDVI is the most accurate when polynomial kernel is used in the regression and the value for constant is fixed at 10 and gamma function is defined as “auto”.

### 5.7. LSTM Regression Analysis

**Figure 11:**
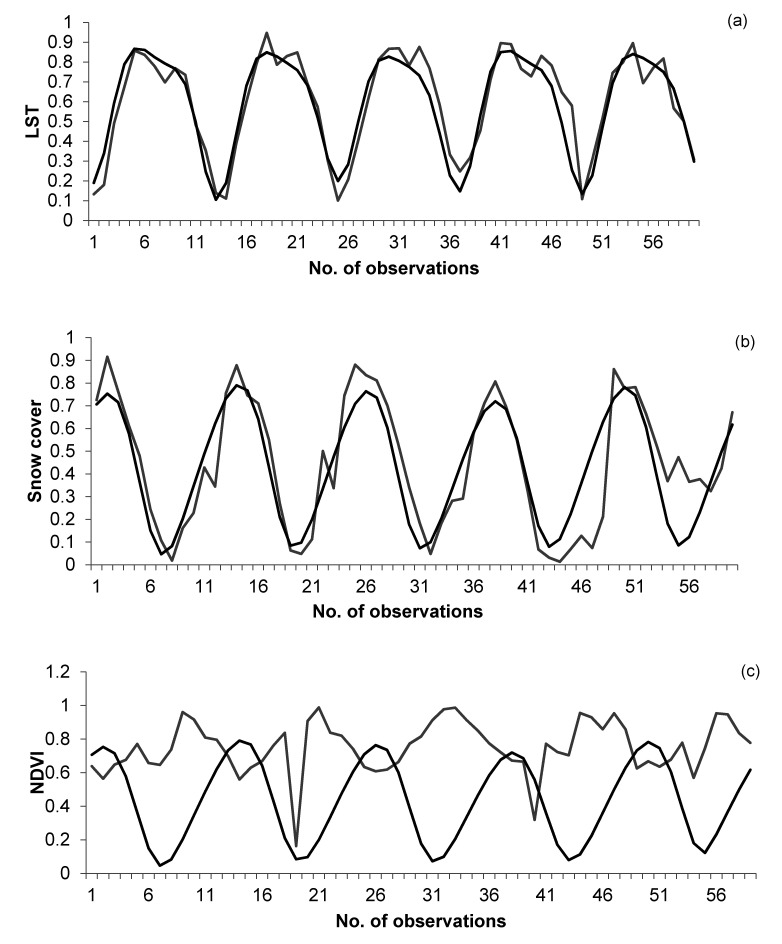
(**a**) Linear curve for the forecasted values of temperature using LSTM networks (black curve) and average values of temperature obtained using MODIS grids (grey curve) for each month for the years 2012–2017 for entire Uttarakhand. (**b**) Linear curve for the forecasted values of snow covers using LSTM networks (black curve) and average values of snow covers obtained using MODIS grids (grey curve) for each month for the years 2012–2017 for entire Uttarakhand. (**c**) Linear curve for the forecasted values of NDVI using LSTM networks (black curve) and average values of NDVI obtained using MODIS grids (grey curve) for each month for the years 2012–2017 for entire Uttarakhand.

Forecasted values of temperature using LSTM networks were compared with the values from MODIS temperature grids for the years 2012–2017 (Figure 11a). Similarly, forecasted values of snow covers using LSTM networks were compared with the values from MODIS snow grids for the years 2012–2017 (Figure 11b) and forecasted values of NDVI using LSTM networks were compared with the values from MODIS NDVI grids for the years 2012–2017 (Figure 11c).

Forecasted values of the climate parameters obtained through LSTM networks and actual values obtained through MODIS grids were compared using regression and correlation. LSTM networks can be considered appropriate for forecasting of temperature (R-squared= 0.87; correlation coefficient = 0.93) and snow cover (R-squared = 0.74; correlation coefficient = 0.86) but not appropriate for forecasting of NDVI (R-squared = 0.20; correlation coefficient = −0.36).

### 5.8. Global Climate Data

**Figure 12:**
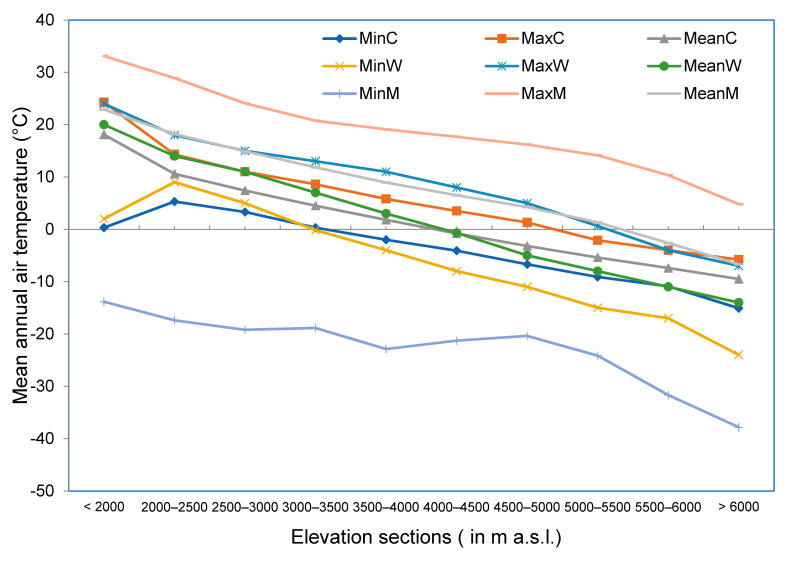
Minimum, maximum and mean values of mean annual air temperature (BIO1) obtained for each elevation section using WorldClim 2.0, CHELSA and MODIS observations. Here, MinC, MaxC and Mean C represent minimum, maximum and mean values of mean annual air temperature obtained for each elevation section using CHELSA. Similarly, MinW, MaxW and MeanW represent minimum, maximum and mean values of mean annual air temperature obtained for each elevation section using WorldClim 2.0. Correspondingly, MinM, MaxM and MeanM represent minimum, maximum and mean values of land surface temperature obtained for each elevation section for the period from 2001 to 2017 using MODIS observations.

Curves indicate that there are significant differences in values among the global climate data set and maximum and minimum values obtained from MODIS LST observations. However, mean values obtained from MODIS are more or less in close agreement with the values obtained from global climate data. Maximum values from WorldClim 2.0 are almost similar to mean values from MODIS LST (Figure 12).

**Figure 13:**
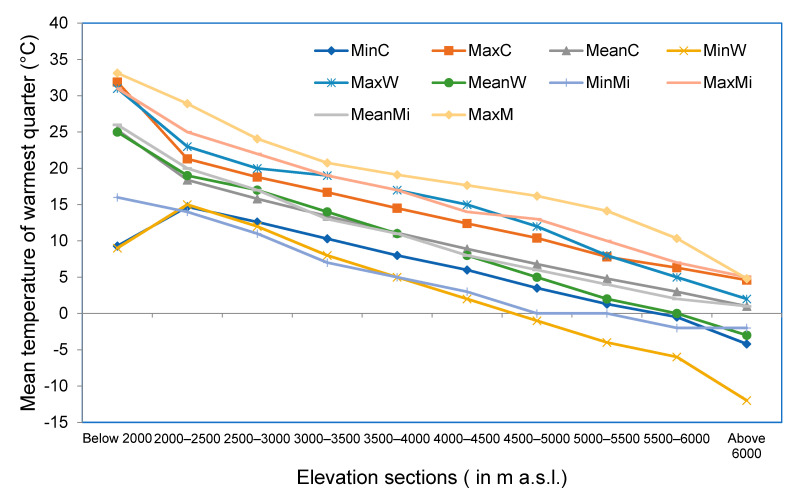
Minimum, maximum and mean values of mean temperature of warmest quarter (BIO10) obtained for each elevation section using WorldClim 2.0, CHELSA and MIROC climate dataset. Here, MinC, MaxC and Mean C represent minimum, maximum and mean values of mean temperature of warmest quarter obtained for each elevation section using CHELSA. Similarly, MinW, MaxW and MeanW represent minimum, maximum and mean values of mean temperature of the warmest quarter obtained for each elevation section using WorldClim 2.0. Correspondingly, MinMi, MaxMi and MeanMi represent minimum, maximum and mean values of mean temperature of warmest quarter obtained for each elevation section using MIROC. Similarly, MaxM represents maximum values of land surface temperature obtained for each elevation section for the period from 2001 to 2017 using MODIS observations.

Curves indicate that maximum values obtained from MODIS observations are higher than values obtained from all other climate data set. Values obtained from the three global climate data are more or less in close agreement with each other (Figure 13).

**Figure 14:**
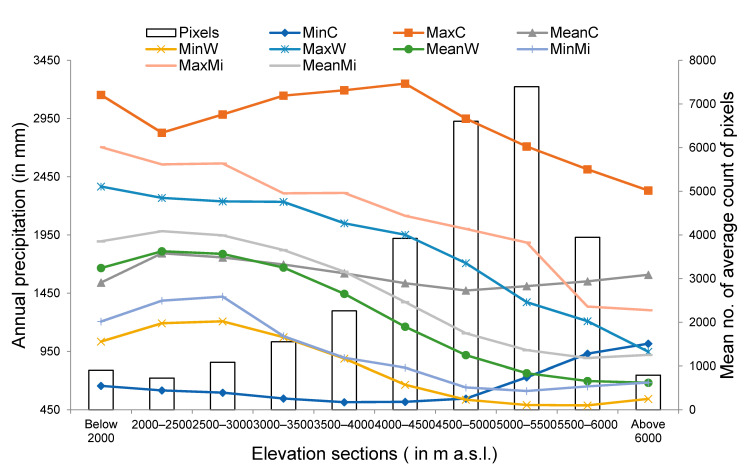
Curves represent minimum, maximum and mean values of annual precipitation obtained for each elevation section using WorldClim 2.0, CHELSA and MIROC climate dataset. Here, MinC, MaxC and Mean C represent minimum, maximum and mean values of annual precipitation obtained for each elevation section using CHELSA. Similarly, MinW, MaxW and MeanW represent minimum, maximum and mean values of annual precipitation obtained for each elevation section using WorldClim 2.0. Correspondingly, MinMi, MaxMi and MeanMi represent minimum, maximum and mean values of annual precipitation obtained for each elevation section using MIROC. Bars represent mean values of average count of pixels representing snow covered area for each elevation section obtained using MODIS observations.

Higher bars are present from 4000 to 6000 m a.s.l., whereas curves steadily report decreasing values of annual precipitation for these ranges (Figure 14). This is mainly because snowfall and, hence, increases in snow-covered areas, are visible above 4000 m a.s.l., whereas annual precipitation is higher in the low elevation ranges below 3500 m a.s.l. Most of the precipitation received by lower elevations is in liquid, while most of annual precipitation above 3500 m a.s.l. remains solid in nature.

**Figure 15:**
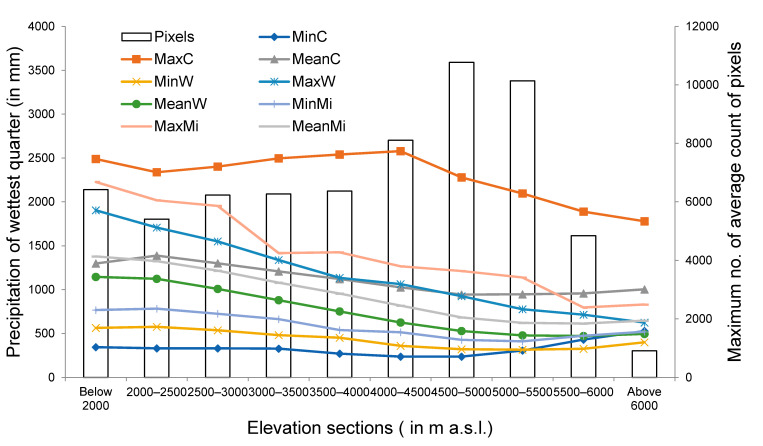
Curves represent minimum, maximum and mean values of precipitation of wettest quarter obtained for each elevation section using WorldClim 2.0, CHELSA and MIROC climate dataset. Here, MinC, MaxC and Mean C represent minimum, maximum and mean values of precipitation of wettest quarter obtained for each elevation section using CHELSA. Similarly, MinW, MaxW and MeanW represent minimum, maximum and mean values of precipitation of wettest quarter obtained for each elevation section using WorldClim 2.0. Correspondingly, MinMi, MaxMi and MeanMi represent minimum, maximum and mean values of precipitation of wettest quarter obtained for each elevation section using MIROC. Bars represent the maximum number of average count of pixels representing snow covered area for each elevation section obtained using MODIS observations.

There is little consistency among the bars representing the maximum number of average count of pixels for different elevation sections and the curves representing the precipitation during the wettest quarter, which mainly confirms that the precipitation during wettest quarter remains mainly liquid in nature in Uttarakhand (Figure 15).

When minimum, maximum and mean values of mean annual air temperature from global climate data were compared against the minimum, maximum and mean values of temperature from MODIS observations, differences were minimum when the mean values were selected (Table 2).

When minimum, maximum and mean values of mean temperature during warmest quarter from global climate data were compared against the minimum, maximum and mean values of temperature from MODIS observations, differences were minimum when the mean and maximum values were selected (Supplementary Table S1). Significant negative associations among mean pixels and some of the climate data simply indicate that with elevation, the number of pixels increases whereas the values for annual precipitation decrease (Supplementary Table S2). This is mainly because annual precipitation is mainly comprised of liquid precipitation in Uttarakhand and the amount of annual precipitation decreases with altitude whereas snow covered areas due to solid precipitation increases with elevation. No significant association exists among the max pixels and the global climate data. Climate data from CHELSA, WorldClim 2.0 and MIROC indicate that MAAT values gradually decrease with an increase in elevation from 2500 m a.s.l. to 4500 m a.s.l. Among the three global climate data, MIROC has the highest values for MAAT whereas CHELSA has the lowest values for MAAT in Uttarakhand (Supplementary Figures S1, S5 and S9). Similarly, climate data from CHELSA, WorldClim 2.0 and MIROC also indicate that T-WQ values gradually decrease with increase in elevation from 2500 m a.s.l. to 4500 m a.s.l. Again, among the three global climate data, MIROC has the highest values for T-WQ whereas CHELSA has the lowest values for T-WQ in Uttarakhand (Supplementary Figures S2, S6 and S10). Climate data from WorldClim 2.0 and MIROC indicate that AnP and P-WQ values gradually decrease with an increase in elevation from 2500 m a.s.l. to 4500 m a.s.l. However, CHELSA shows higher values of AnP and P-WQ when the elevation increases (Supplementary Figures S3, S4 and S7, Supplementary Figures S8, S11 and S12).

## 6. Discussion

A previous investigation [44] mentioned the decrease in snowfall in Uttarakhand for the years 1991–2015. Present investigation reports an increase in annual snow covers for the years 2001–2017 and this is particularly visible for the pre-monsoon period.

The Indian Institute of Remote Sensing recently published a report detailing an analysis of snow extents for the Uttarakhand state, which stated that highest snowfall occurred in 2016 and 2017 [45]. The year 2017 had the highest snowfall when in the month of February; snow covers persisted in almost 67% of areas in the north-west Himalayas. If we consider this finding, the results reported from the present study using MODIS data set are in close agreement with this report.

Snowfall records for the months November–April from 2002 to 2008, available from a weather station in Uttarakhand, was compared with the sum of grid cells representing snow covers within entire Uttarakhand for the months November-April from 2002 to 2008. Coefficient of determination thus obtained was about 0.75. This fairly suggests that information about snow covers from MODIS grids reasonably exemplifies snowfall events in the north-west part of Himalayan region.

Results related to changes in NDVI from the present investigation closely correspond to outcomes obtained from past studies that document analogous negative trends related to NDVI parameters for the Uttarakhand state through analysis of MODIS grids [46]. Similarly, results related to changes in snow covers from the present investigation also closely correspond to outcomes obtained from past studies that document analogous increasing trends related to snow covers for the Uttarakhand state through analysis of MODIS grids [10].

Maps of monthly variations of environmental variables in 2017 (Figure 5, Figure 6 and Figure 7) and monthly lapse rate (Figure 10) indicate a strong seasonal influence upon climate variables. Maps and graphical representations in this study highlight the importance of visual analysis in the interpretation of environmental changes on a regional level.

Application of machine learning algorithms to bulk processed remotely sensed data set is attainable only through efficient computations using proper infrastructures. The present investigation is a good example of the study of environmental variables in the Himalayan region. However, the study is confined to a single state in India. Wide-ranging investigations of this nature can be very illustrative and contribute towards a stronger knowledge base related to climate variables and their changes over time. Nevertheless, this is attainable only through a larger amount of processed data sets and much more complex computations.

Mean temperature values obtained from MODIS are in greater agreement with the global climate data and therefore, for future assessments, mean MODIS values could be used for effective comparison with output from global climate data when these data are used for environmental modelling in the region. Reasonable association among the snow cover area information from MODIS observations and the precipitation information from global climate data cannot be found and therefore, this combination of data set is not appropriate to be used together for precipitation assessment and additional snowfall records from other sources are necessary to establish a meaningful relationship among the data set. WorldClim 2.0 and MIROC serve as better precipitation data sets for any kind of environmental modelling, since CHELSA predicts increasing precipitation values with an increase in elevation, which is highly unlikely.

MODIS grids are available after 2017 as well and therefore, the study could have been extended to 2020, thus completing two decades of analysis and results. However, this study only consists of observations and results for the years 2001–2017 to halt data download and proceed with the analysis of the downloaded data to obtain desired outcomes. A total of 17 years of data are used for analysis, which fairly represents the variations of environmental parameters over nearly two decades. A greater interest of this study lies in experimenting with several methods to analyse the available information for 17 years through multiple MODIS grids.

Although several stations could have been included, records from only two meteorological stations are considered to compare with the remotely sensed information using MODIS.

## 7. Conclusions

The outcomes of this study are essential in formulating assumptions for investigations related to modelling of environmental components and processes in the study area. Results from temperature lapse rate analysis were found to be essential in developing models associated with glacier melt, snow melt and other processes which use temperature as an important input parameter. Results also corroborated that MODIS data set in combination with global climate data set can be used to study past, present and future climate conditions. The past and future climate data set from WorldClim 2.0 proves to be a better choice while working with global climate data sets.

Although this study reasonably captures the variability in climate parameters since 2001, inclusion of climate data set exceeding larger time scales would have been essential in understanding changes over several decades and not just the recent past. Despite that machine learning algorithms have been implemented to understand the changes in and interconnectedness among climate variables, there were limited data available for the training and testing of the algorithms. Climate data expanding several decades would have been more convenient to work with these algorithms.

Similar studies in the future could contribute towards a deeper understanding of environmental changes in the region assisting several stakeholders. Future research could consider adding precipitation, evapotranspiration, as well as other variables, to correlate additional variables with the parameters already under consideration in this study.

## Figures and Tables

**Table 1:** Summary of the remotely sensed data and field-based observations used in this study (NA refers to not applicable).

**Remote Sensing Data**			
**Grids**		**Grid Cell Size**	**Temporal Resolution**
MOD10A2		500 m	8 days
MOD11A2		1000 m	8 days
MOD13A1		500 m	16 days
SRTMGL1 V003		30 m	NA
WorldClim 2.0 (BIO1, BIO10, BIO12 and BIO16)		1000 m	NA
CHELSA (BIO1, BIO10, BIO12 and BIO16)		1000 m	NA
MIROC-ES2L (BIO1, BIO10, BIO12 and BIO16)		1000 m	NA
**Meteorological Records**			
**Station**	**Duration**	**Type**	
Mukhem	2001–2008	Average value of maximum temperature for each month	
Kausani	2001–2009	Average value of maximum temperature for each month	

**Table 2:** Differences among MODIS land surface temperature observations and BIO1 values obtained from different climate data set. Here, MinC, MaxC and Mean C represent minimum, maximum and mean values of mean annual air temperature obtained for each elevation section using CHELSA. Similarly, MinW, MaxW and MeanW represent minimum, maximum and mean values of mean annual air temperature obtained for each elevation section using WorldClim 2.0. Correspondingly, MinM, MaxM and MeanM represent minimum, maximum and mean values of mean annual air temperature obtained for each elevation section using MODIS observations. In the same way, MinMi, MaxMi and MeanMi represent minimum, maximum and mean values of mean annual air temperature obtained for each elevation section using MIROC.

Elevation Range (m a.s.l.)	MinM-MinC	MaxM-MaxC	MeanM-MeanC	MinM-MinW	MaxM-MaxW	MeanM-MeanW	MinM-MinMi	MaxM-MaxMi	MeanM-MeanMi
<2000	−14	9	5	−16	9	3	−25	8	3
2000–2500	−23	15	8	−26	11	4	−26	9	3
2500–3000	−23	13	8	−24	9	4	−24	7	3
3000–3500	−19	12	7	−19	8	5	−19	6	4
3500–4000	−21	13	7	−19	8	6	−21	7	4
4000–4500	−17	14	7	−13	10	7	−17	9	5
4500–5000	−14	15	7	−9	11	9	−12	8	4
5000–5500	−15	16	7	−9	14	9	−16	10	4
5500–6000	−21	14	5	−15	14	8	−21	10	2
>6000	−23	11	3	−14	12	7	−27	6	−1

## Supplementary Materials

The following are available online at https://www.mdpi.com/article/10.3390/s21217416/s1. Figure S1: BIO1 or MAAT values recorded using CHELSA climate data set for the elevation sections (a) 2500–3000 m a.s.l., (b) 3000–3500 m a.s.l., (c) 3500–4000 m a.s.l. and (d) 4000–4500 m a.s.l., Figure S2: BIO10 or T-WQ values recorded using CHELSA climate data set for the elevation sections (a) 2500–3000 m a.s.l., (b) 3000–3500 m a.s.l., (c) 3500–4000 m a.s.l. and (d) 4000–4500 m a.s.l., Figure S3: BIO12 or AnP values recorded using CHELSA climate data set for the elevation sections (a) 2500–3000 m a.s.l., (b) 3000–3500 m a.s.l., (c) 3500–4000 m a.s.l. and (d) 4000–4500 m a.s.l., Figure S4: BIO16 or P-WQ values recorded using CHELSA climate data set for the elevation sections (a) 2500–3000 m a.s.l., (b) 3000–3500 m a.s.l., (c) 3500–4000 m a.s.l. and (d) 4000–4500 m a.s.l., Figure S5: BIO1 or MAAT values recorded using WorldClim 2.0 climate data set for the elevation sections (a) 2500–3000 m a.s.l., (b) 3000–3500 m a.s.l., (c) 3500–4000 m a.s.l. and (d) 4000–4500 m a.s.l., Figure S6: BIO10 or T-WQ values recorded using WorldClim 2.0 climate data set for the elevation sections (a) 2500–3000 m a.s.l., (b) 3000–3500 m a.s.l., (c) 3500–4000 m a.s.l. and (d) 4000–4500 m a.s.l., Figure S7: BIO12 or AnP values recorded using WorldClim 2.0 climate data set for the elevation sections (a) 2500–3000 m a.s.l., (b) 3000–3500 m a.s.l., (c) 3500–4000 m a.s.l. and (d) 4000–4500 m a.s.l., Figure S8: BIO16 or P-WQ values recorded using WorldClim 2.0 climate data set for the elevation sections (a) 2500–3000 m a.s.l., (b) 3000–3500 m a.s.l., (c) 3500–4000 m a.s.l. and (d) 4000–4500 m a.s.l., Figure S9: BIO1 or MAAT values recorded using MIROC climate data set for the elevation sections (a) 2500–3000 m a.s.l., (b) 3000–3500 m a.s.l., (c) 3500–4000 m a.s.l. and (d) 4000–4500 m a.s.l., Figure S10: BIO10 or T-WQ values recorded using MIROC climate data set for the elevation sections (a) 2500–3000 m a.s.l., (b) 3000–3500 m a.s.l., (c) 3500–4000 m a.s.l. and (d) 4000–4500 m a.s.l., Figure S11: BIO12 or AnP values recorded using MIROC climate data set for the elevation sections (a) 2500–3000 m a.s.l., (b) 3000–3500 m a.s.l., (c) 3500–4000 m a.s.l. and (d) 4000–4500 m a.s.l., Figure S12: BIO16 or P-WQ values recorded using MIROC climate data set for the elevation sections (a) 2500–3000 m a.s.l., (b) 3000–3500 m a.s.l., (c) 3500–4000 m a.s.l. and (d) 4000–4500 m a.s.l. Table S1: Differences among MODIS land surface temperature observations and BIO10 values obtained from different climate data set, Table S2: Pearson correlation coefficients among BIO12 values obtained from climate data set and mean values of average count of pixels representing snow covered area for each elevation section obtained using MODIS observations.

## Abbreviations

MODIS	moderate resolution imaging spectroradiometer
LSTM	long short-term memory
LST	land surface temperature
NDVI	normalized difference vegetation index
SRTM	shuttle radar topography mission
CHELSA	climatologies at high resolution for the earth’s land surface areas
MIROC-ES2L	model for interdisciplinary research on climate, earth system version 2 for long-term simulations
HDF	hierarchical data format
HTTPS	hyper text transfer protocol secure
TIFF	tag image file format
CSV	comma-separated values
IBM SPSS	international business machines corporation’s statistical package for the social sciences
