# Supplementary material for: Bulk Processing of Multi-Temporal Modis Data, Statistical Analyses and Machine Learning Algorithms to Understand Climate Variables in the Indian Himalayan Region
Source: Sensors (Basel). 2021 Nov 8;21(21):7416. doi: 10.3390/s21217416 (PMC8588406; doi:10.3390/s21217416)
Supplement: Supplementary file 1 [file sensors-21-07416-s001.zip › sensors-1389205-support.pdf]

sensors

MDPI

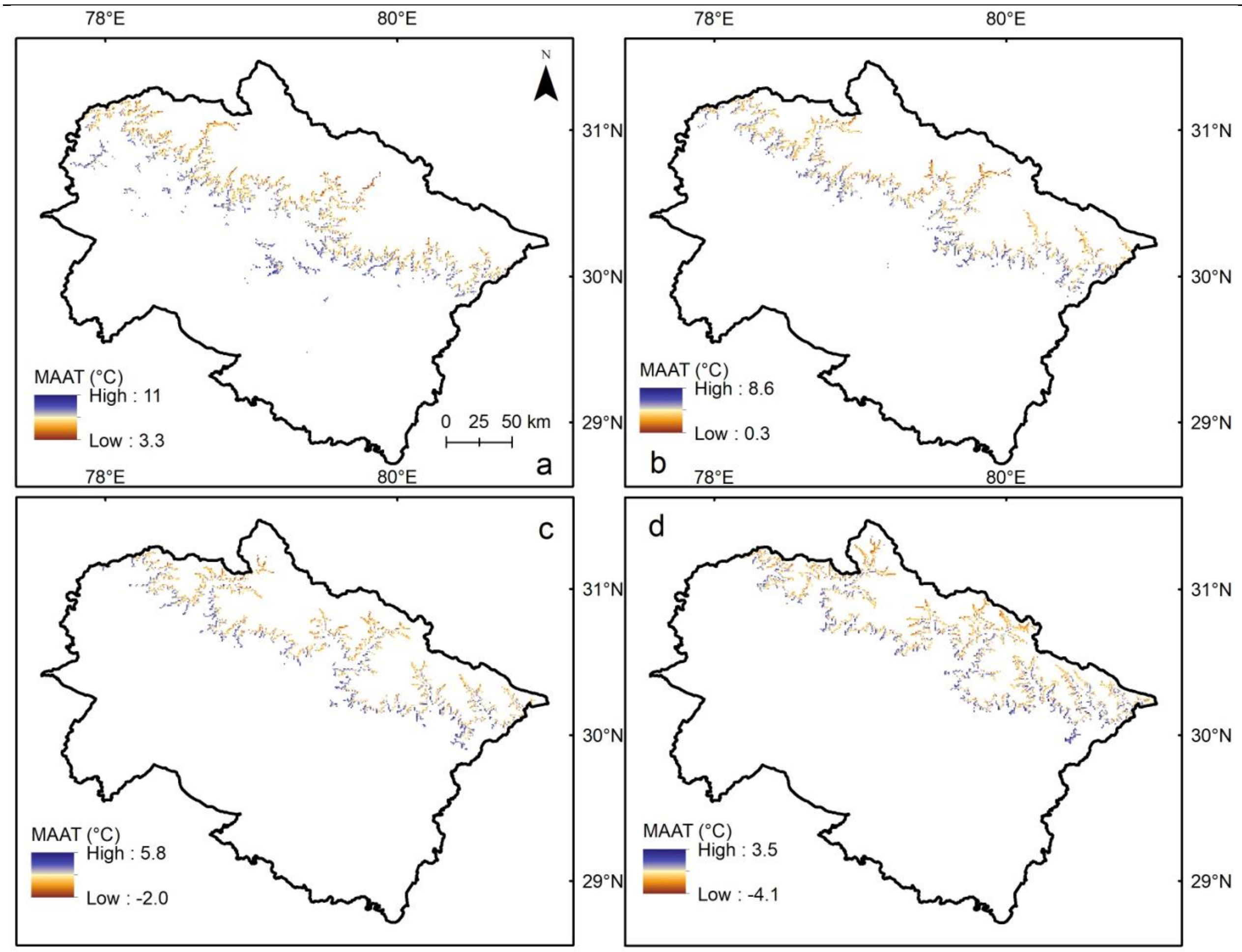

Supplementary Figure S1. BIO1 or MAAT values recorded using CHELSA climate data set for the elevation sections (**a**) 2500–3000 m a.s.l., (**b**) 3000–3500 m a.s.l., (**c**) 3500–4000 m a.s.l. and (**d**) 4000–4500 m a.s.l.

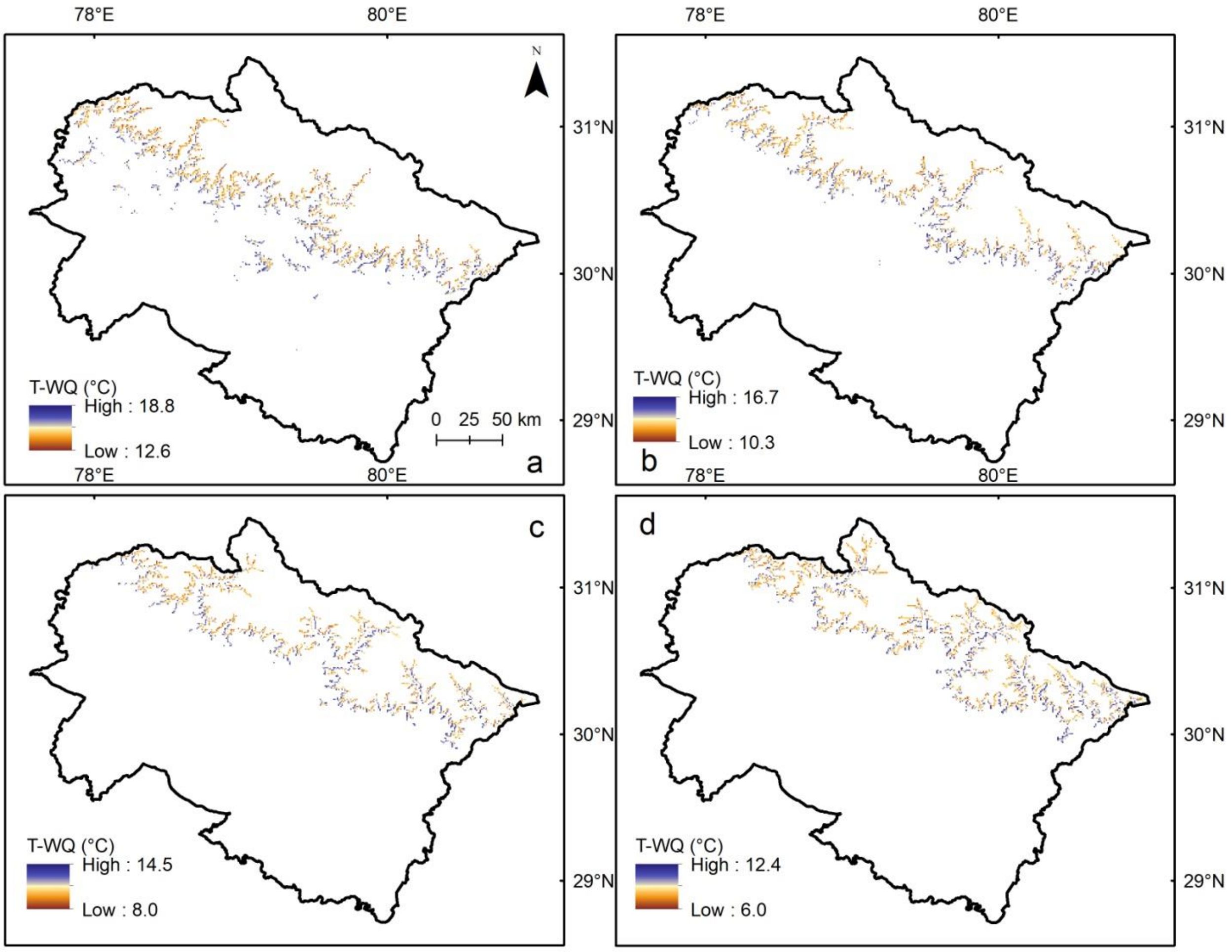

Supplementary Figure S2. BIO10 or T-WQ values recorded using CHELSA climate data set for the elevation sections (**a**) 2500–3000 m a.s.l., (**b**) 3000–3500 m a.s.l., (**c**) 3500–4000 m a.s.l. and (**d**) 4000–4500 m a.s.l.

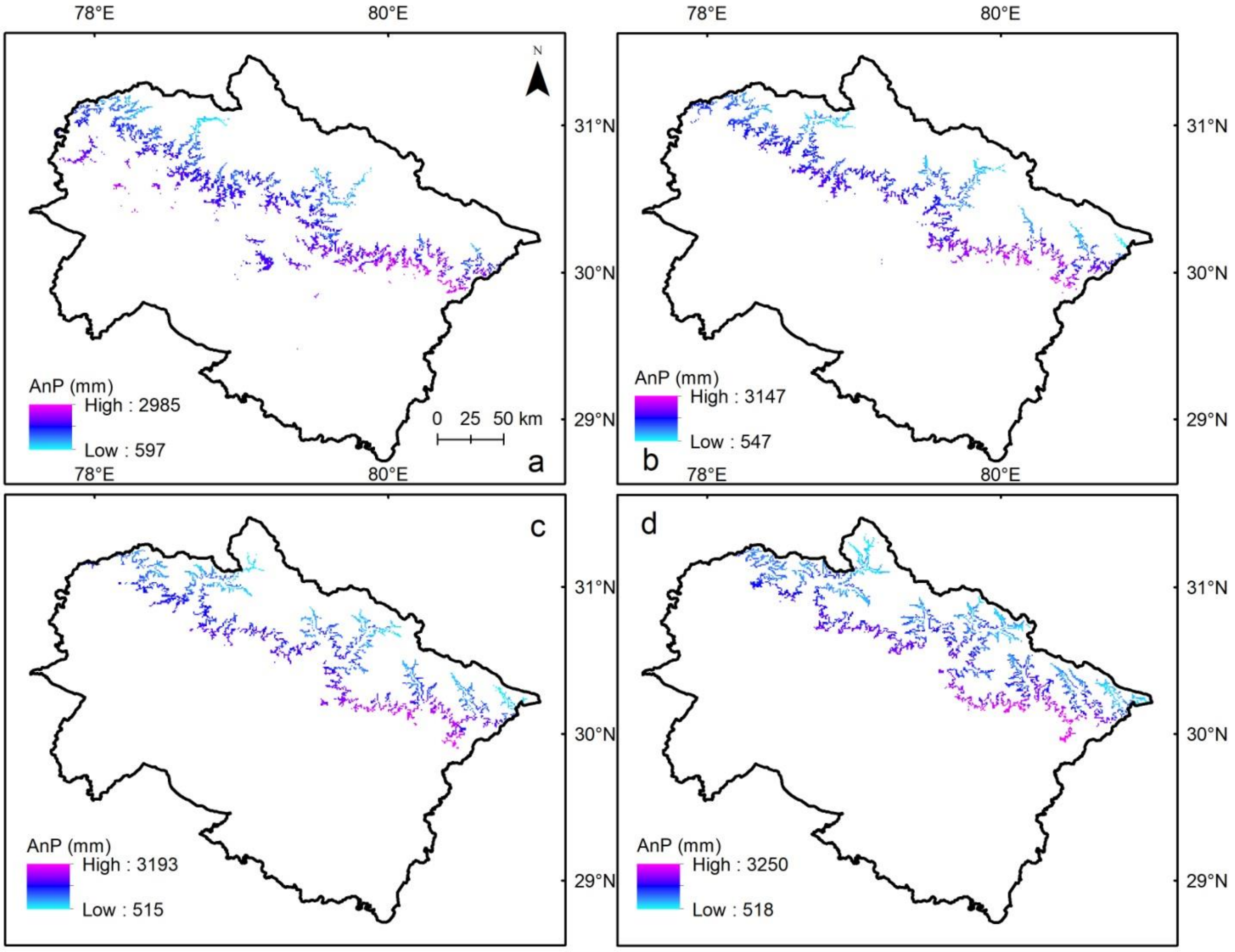

Supplementary Figure S3. BIO12 or AnP values recorded using CHELSA climate data set for the elevation sections (**a**) 2500–3000 m a.s.l., (**b**) 3000–3500 m a.s.l., (**c**) 3500–4000 m a.s.l. and (**d**) 4000–4500 m a.s.l.

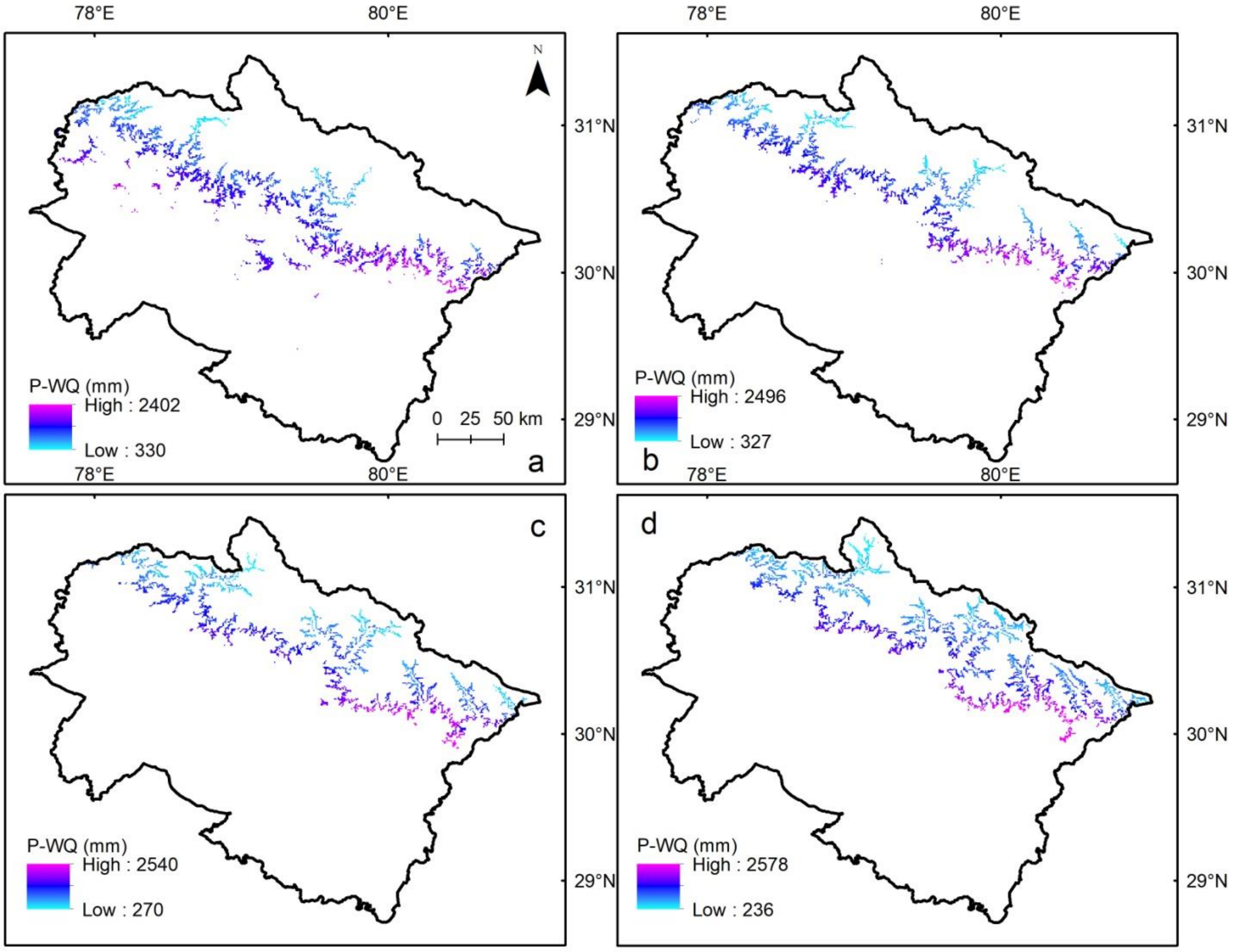

Supplementary Figure S4. BIO16 or P-WQ values recorded using CHELSA climate data set for the elevation sections (**a**) 2500–3000 m a.s.l., (**b**) 3000–3500 m a.s.l., (**c**) 3500–4000 m a.s.l. and (**d**) 4000–4500 m a.s.l.

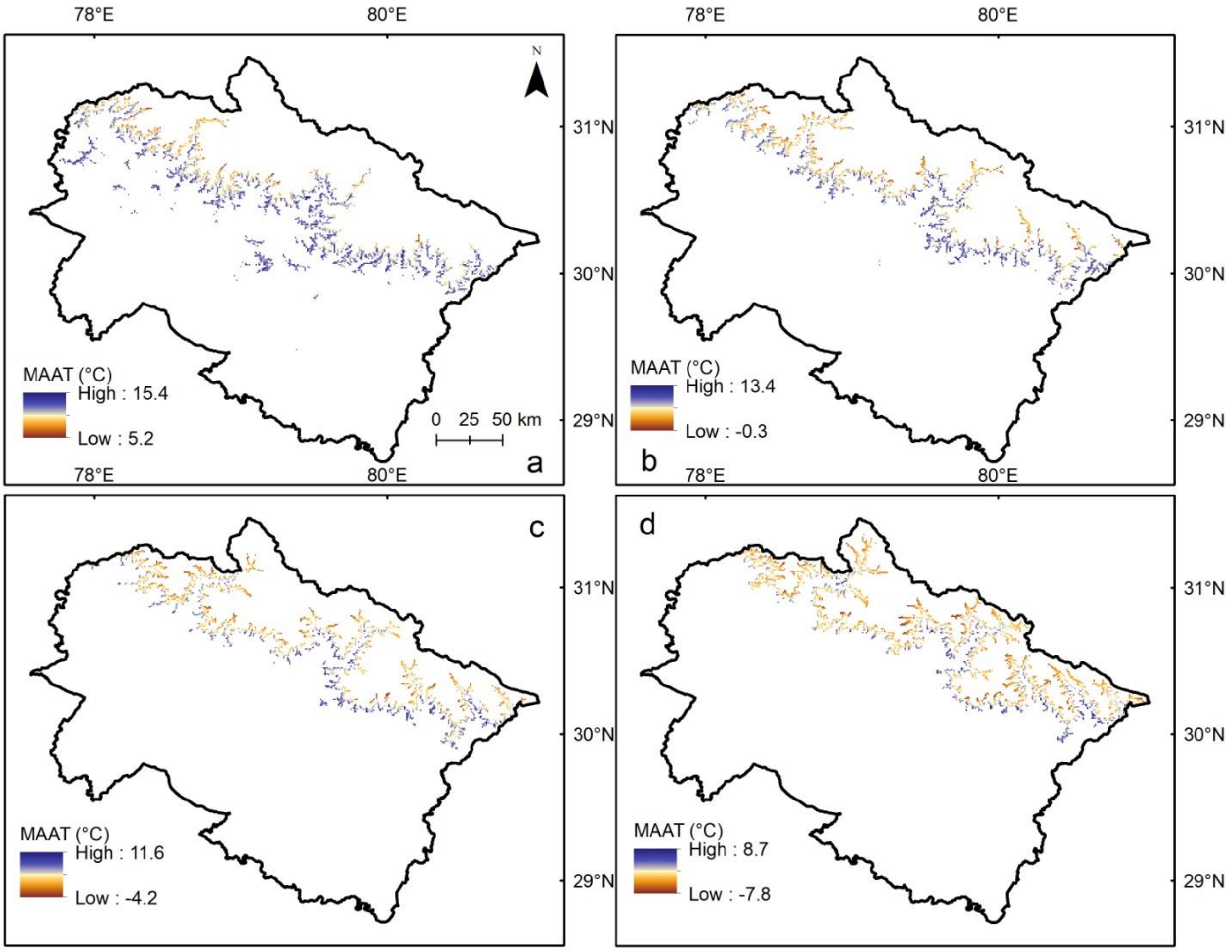

Supplementary Figure S5. BIO1 or MAAT values recorded using WorldClim 2.0 climate data set for the elevation sections (**a**) 2500–3000 m a.s.l., (**b**) 3000–3500 m a.s.l., (**c**) 3500–4000 m a.s.l. and (**d**) 4000–4500 m a.s.l.

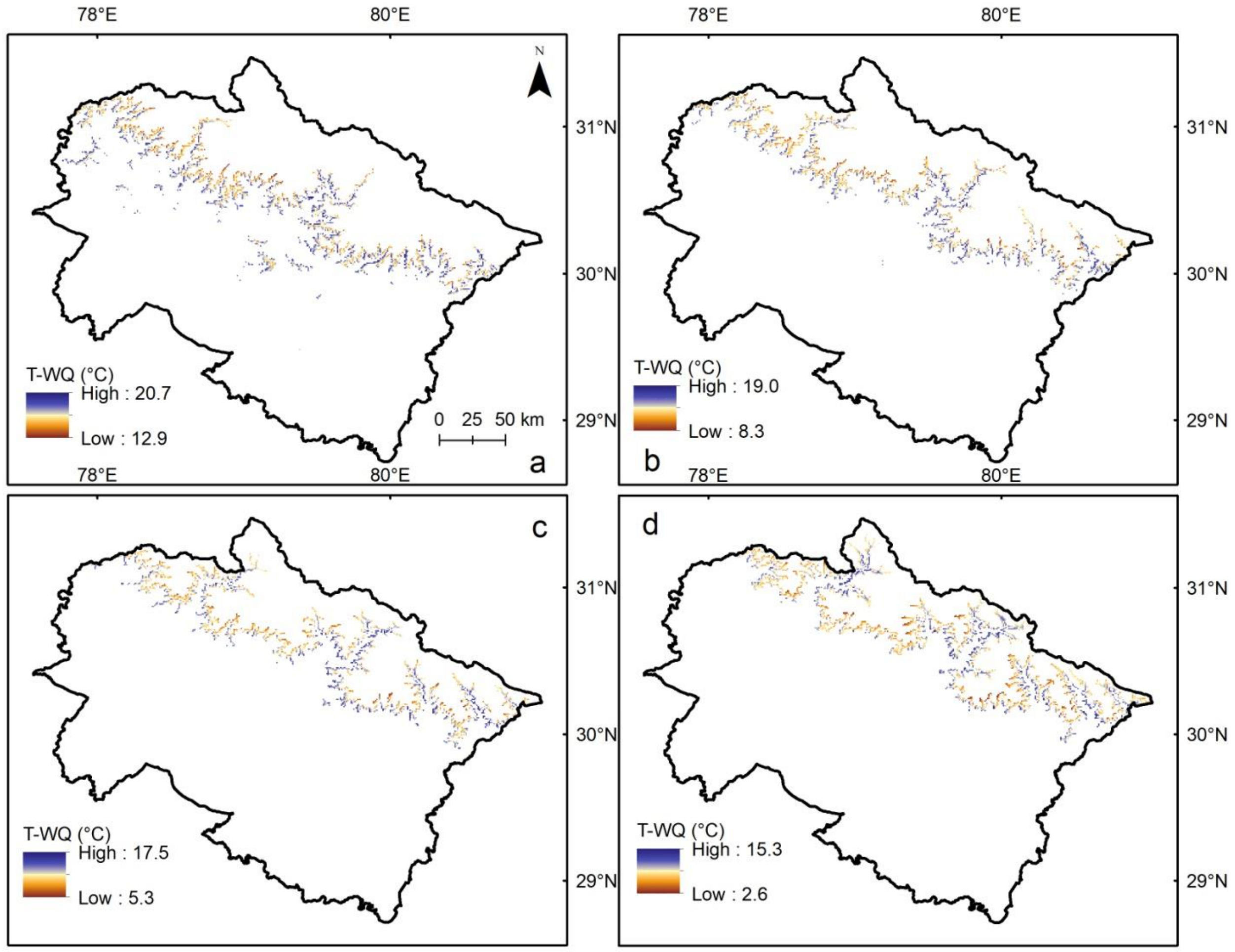

Supplementary Figure S6. BIO10 or T-WQ values recorded using WorldClim 2.0 climate data set for the elevation sections (**a**) 2500–3000 m a.s.l., (**b**) 3000–3500 m a.s.l., (**c**) 3500–4000 m a.s.l. and (**d**) 4000–4500 m a.s.l.

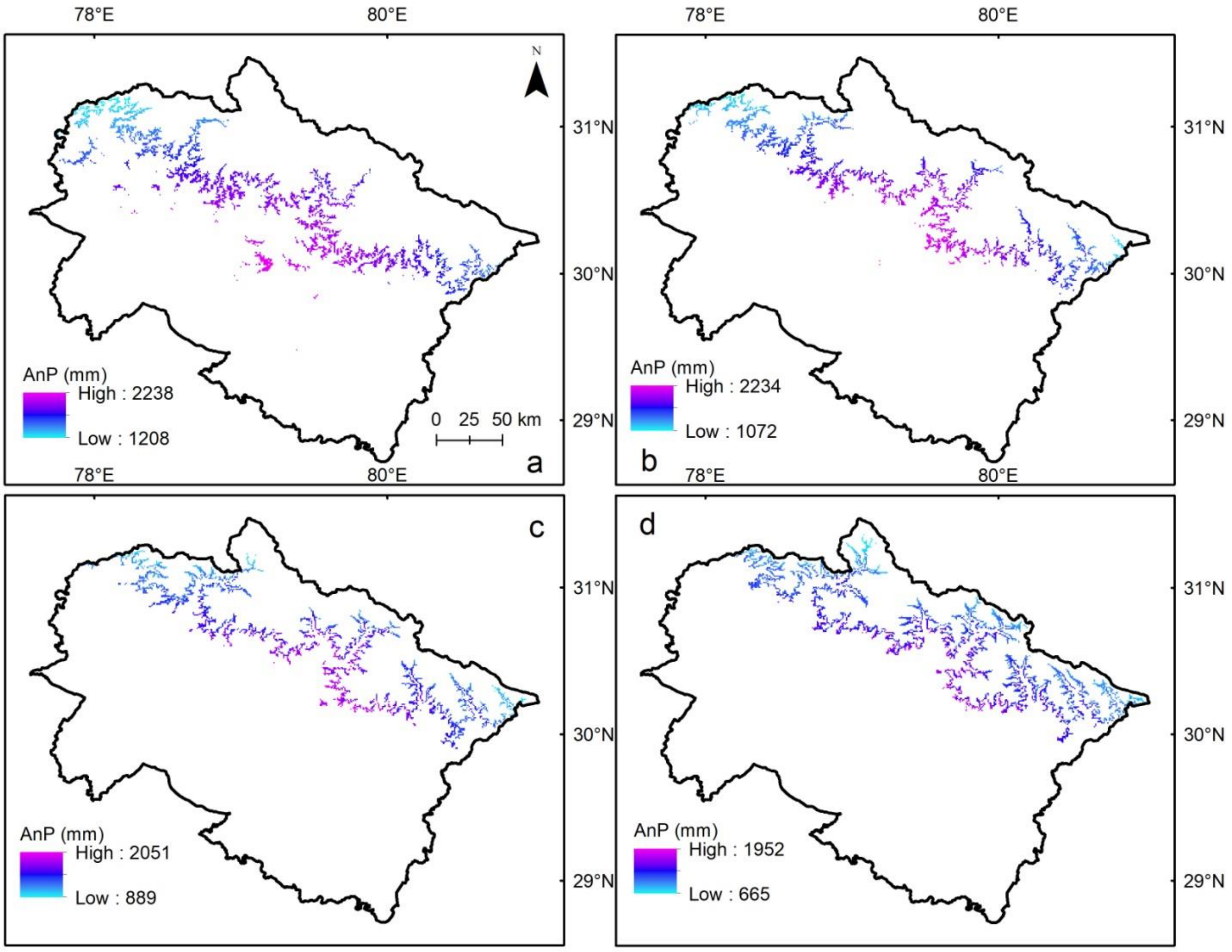

Supplementary Figure S7. BIO12 or AnP values recorded using WorldClim 2.0 climate data set for the elevation sections (**a**) 2500–3000 m a.s.l., (**b**) 3000–3500 m a.s.l., (**c**) 3500–4000 m a.s.l. and (**d**) 4000–4500 m a.s.l.

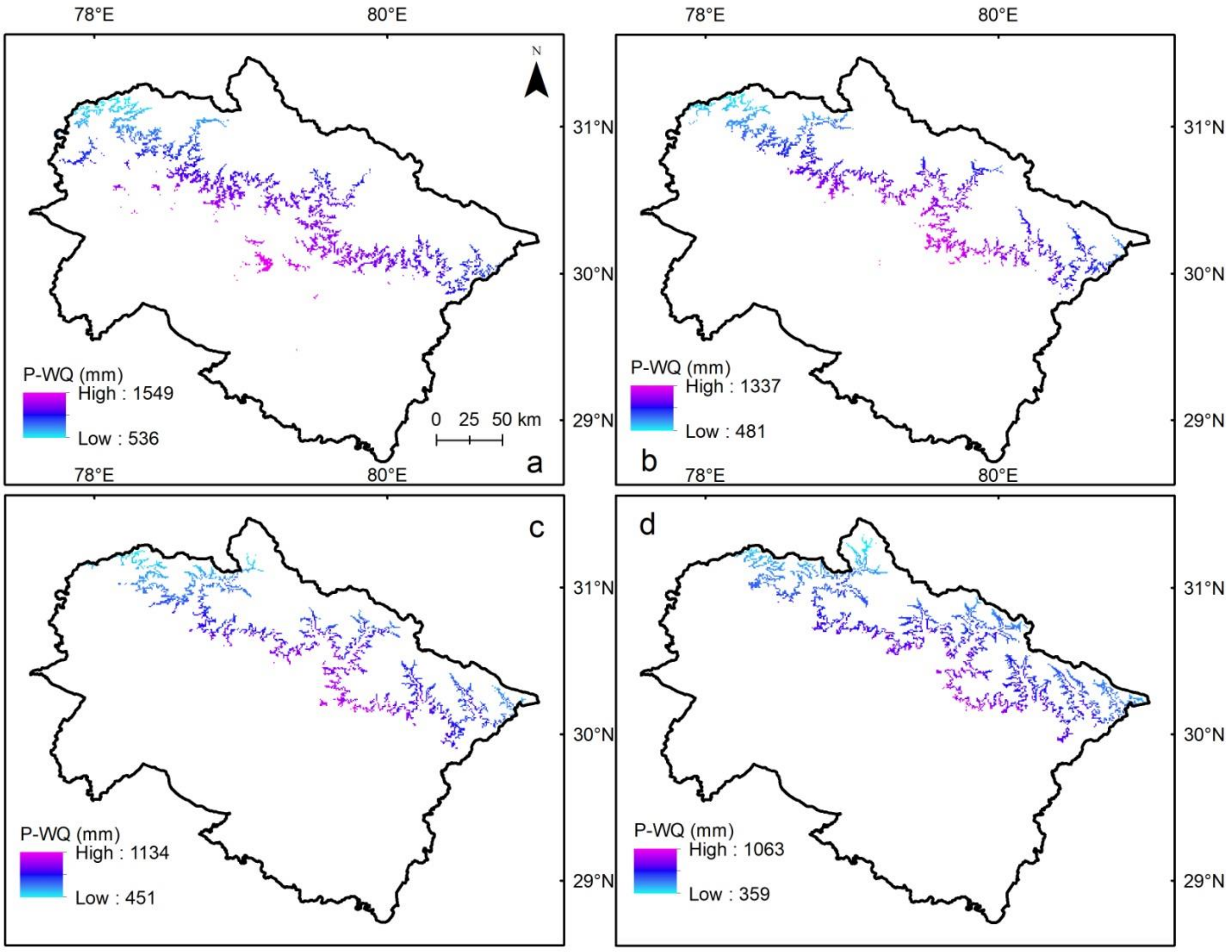

Supplementary Figure S8. BIO16 or P-WQ values recorded using WorldClim 2.0 climate data set for the elevation sections (**a**) 2500–3000 m a.s.l., (**b**) 3000–3500 m a.s.l., (**c**) 3500–4000 m a.s.l. and (**d**) 4000–4500 m a.s.l.

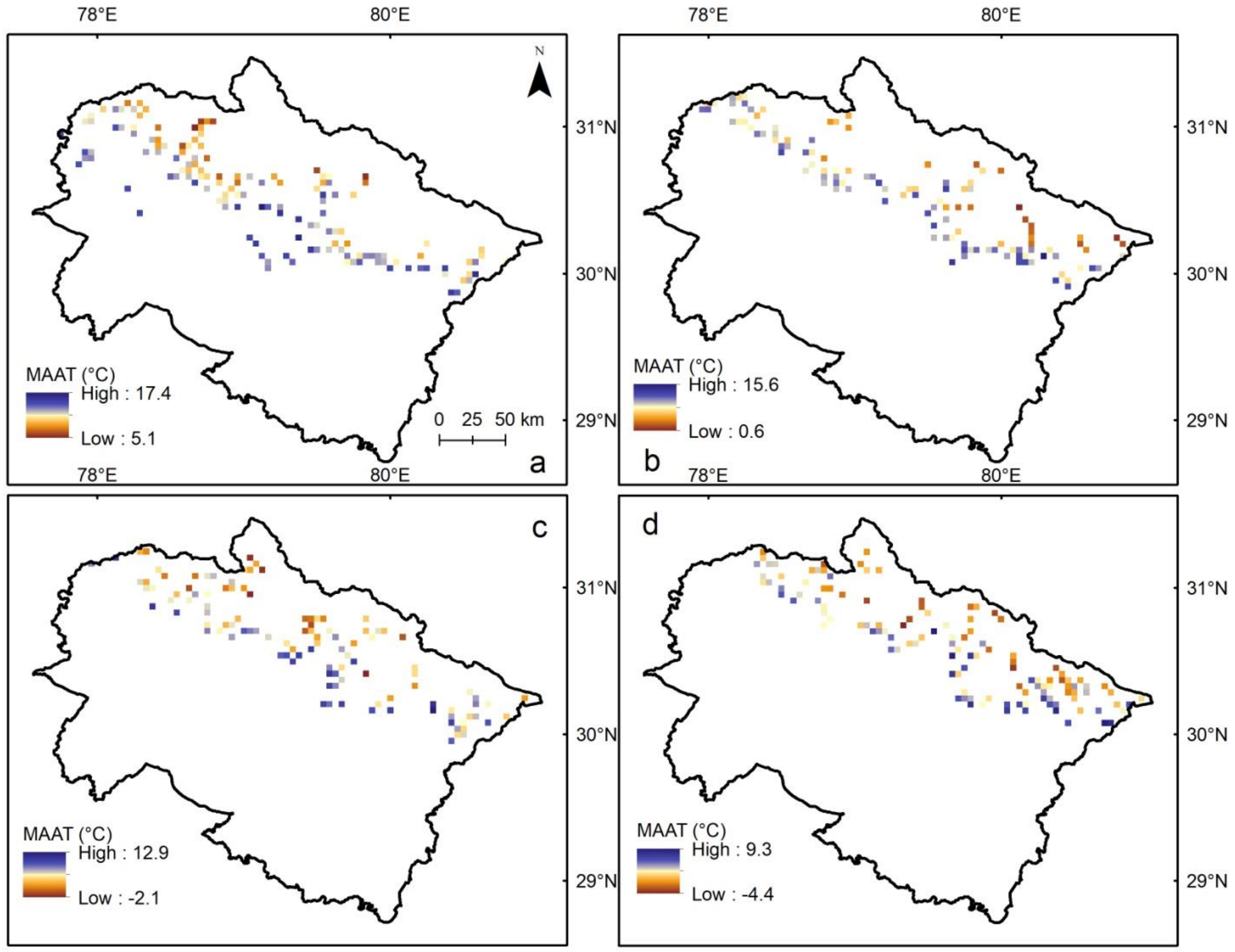

Supplementary Figure S9. BIO1 or MAAT values recorded using MIROC climate data set for the elevation sections (**a**) 2500–3000 m a.s.l., (**b**) 3000–3500 m a.s.l., (**c**) 3500–4000 m a.s.l. and (**d**) 4000–4500 m a.s.l.

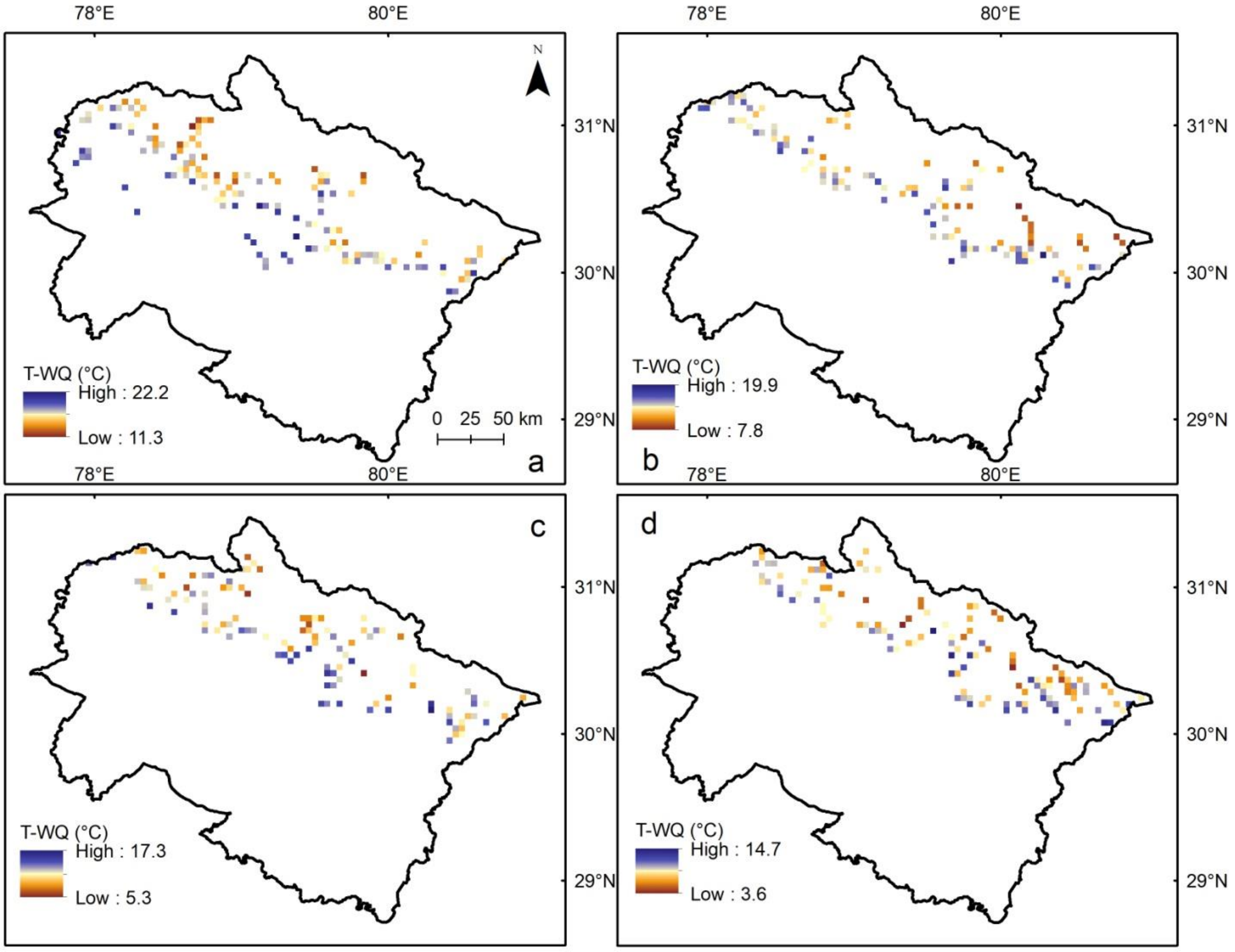

Supplementary Figure S10. BIO10 or T-WQ values recorded using MIROC climate data set for the elevation sections (**a**) 2500–3000 m a.s.l., (**b**) 3000–3500 m a.s.l., (**c**) 3500–4000 m a.s.l. and (**d**) 4000–4500 m a.s.l.

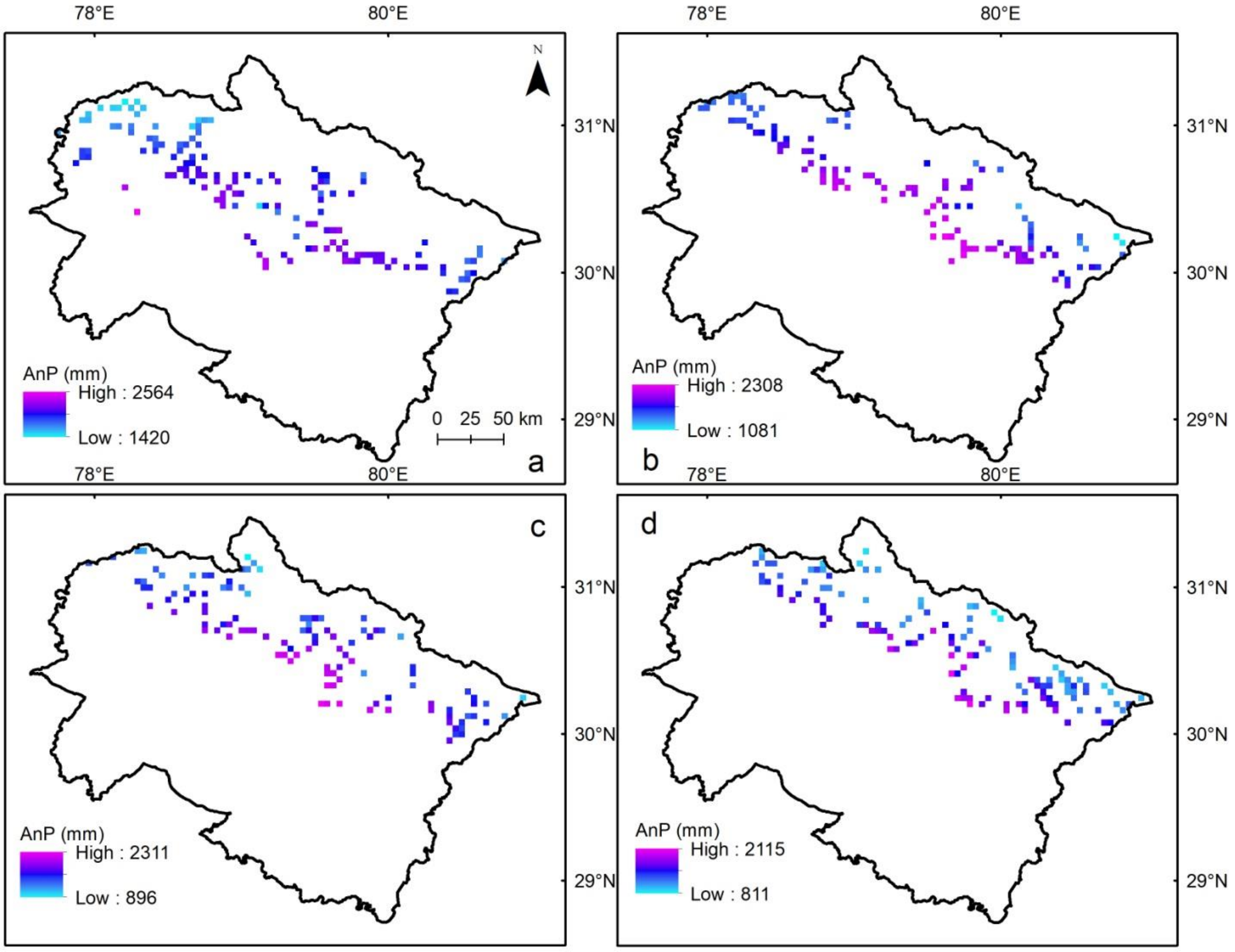

Supplementary Figure S11. BIO12 or AnP values recorded using MIROC climate data set for the elevation sections (**a**) 2500–3000 m a.s.l., (**b**) 3000–3500 m a.s.l., (**c**) 3500–4000 m a.s.l. and (**d**) 4000–4500 m a.s.l.

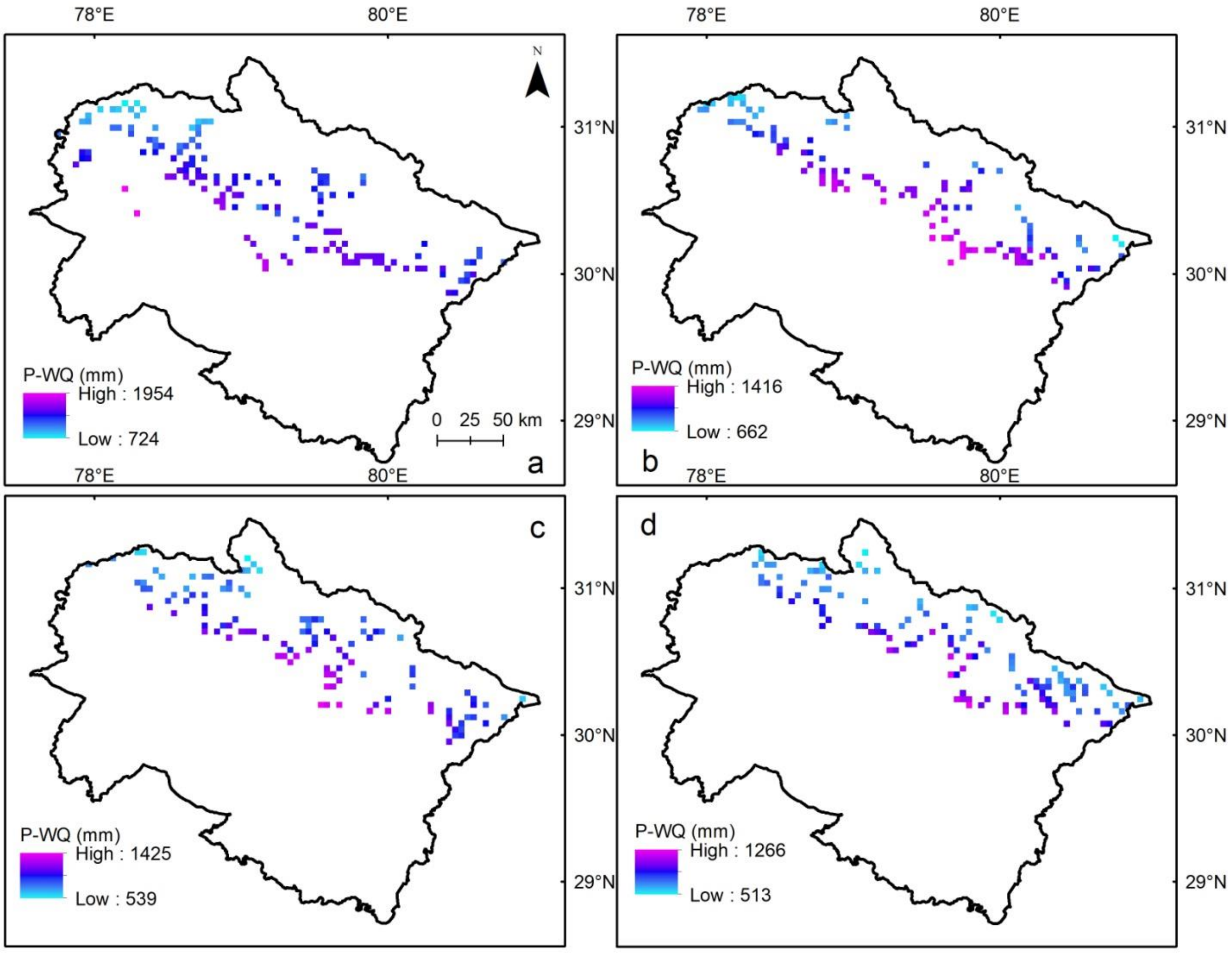

Supplementary Figure S12. BIO16 or P-WQ values recorded using MIROC climate data set for the elevation sections (**a**) 2500–3000 m a.s.l., (**b**) 3000–3500 m a.s.l., (**c**) 3500–4000 m a.s.l. and (**d**) 4000–4500 m a.s.l.

Supplementary Table S1. Differences among MODIS land surface temperature observations and BIO10 values obtained from different climate data set. Here, MinC, MaxC and Mean C represent minimum, maximum and mean values of mean temperature of warmest quarter obtained for each elevation section using CHELSA. Similarly, MinW, MaxW and MeanW represent minimum, maximum and mean values of mean temperature of warmest quarter obtained for each elevation section using WorldClim 2.0. Correspondingly, MinM, MaxM and MeanM represent minimum, maximum and mean values of mean annual air temperature obtained for each elevation section using MODIS observations. In the same way, MinMi, MaxMi and MeanMi represent minimum, maximum and mean values of mean temperature of warmest quarter obtained for each elevation section using MIROC.

Elevation range	MinM-MinC	MaxM-MaxC	MeanM-MeanC	MinM-MinW	MaxM-MaxW	MeanM-MeanW	MinM-MinMi	MaxM-MaxMi	Mea
Below 2000	-23	1	-2	-23	2	-2	-30	2	-3
2000-2500	-32	8	0	-32	6	-1	-31	4	-2
2500-3000	-32	5	-1	-31	4	-2	-30	2	-2
3000-3500	-29	4	-2	-27	2	-2	-26	2	-1
3500-4000	-31	5	-2	-28	2	-2	-28	2	-2
4000-4500	-27	5	-2	-23	3	-1	-24	4	-1
4500-5000	-24	6	-3	-19	4	-1	-20	3	-2
5000-5500	-25	6	-3	-20	6	-1	-24	4	-3
5500-6000	-31	4	-6	-26	5	-3	-30	3	-5
Above 6000	-34	0	-8	-26	3	-4	-36	0	-8

Supplementary Table S2 Pearson correlation coefficients among BIO12 values obtained from climate data set and mean values of average count of pixels representing snow covered area for each elevation section obtained using MODIS observations. Here, MinC, MaxC and Mean C represent minimum, maximum and mean values of annual precipitation obtained for each elevation section using CHELSA. Similarly, MinW, MaxW and MeanW represent minimum, maximum and mean values of annual precipitation obtained for each elevation section using WorldClim 2.0. Correspondingly, MinMi, MaxMi and MeanMi represent minimum, maximum and mean values of annual precipitation obtained for each elevation section using MIROC. Mean pixels represent mean values of average count of pixels representing snow-covered area for each elevation section obtained using MODIS observations.

Pearson correlation coefficients among BIO16 values obtained from climate data set and maximum number of average count of pixels representing snow covered area for each elevation section obtained using MODIS observations. Here, MinC, MaxC and Mean C represent minimum, maximum and mean values of precipitation of wettest quarter obtained for each elevation section using CHELSA. Similarly, MinW, MaxW and MeanW represent minimum, maximum and mean values of precipitation of wettest quarter obtained for each elevation section using WorldClim 2.0. Correspondingly, MinMi, MaxMi and MeanMi represent minimum, maximum and mean values of precipitation of wettest quarter obtained for each elevation section using MIROC. Max pixels represent the maximum number of average count of pixels representing snow covered area for each elevation section obtained using MODIS observations. Bold values indicate statistically significant outputs.

**Bio12**	**MinC**	**MaxC**	**MeanC**	**MinW**	**MaxW**	**MeanW**	**MinMi**	**MaxMi**	**MeanMi**	**Mean**
**Mean**	-0.068	-0.08	**-0.724**	-0.721	-0.416	**-0.636**	**-0.72**	-0.359	**-0.654**	1
**Bio16**	**MinC**	**MaxC**	**MeanC**	**MinW**	**MaxW**	**MeanW**	**MinMi**	**MaxMi**	**MeanMi**	**Max pixels**
**Max pixels**	**-0.86**	0.438	-0.235	-0.34	0.034	-0.116	-0.329	0.106	-0.111	1
